# Standardization of Questions in Rare Disease Registries: The PRISM Library Project

**DOI:** 10.2196/ijmr.2107

**Published:** 2012-10-10

**Authors:** Rachel Lynn Richesson, Denise Shereff, James Everett Andrews

**Affiliations:** 1 Duke University School of NursingDurham, NCUnited States; 2 University of South Florida (USF) College of MedicineDivision of Bioinformatics and BiostatisticsTampa, FLUnited States; 3 School of InformationUniversity of South FloridaTampa, FLUnited States

**Keywords:** Patient registries, data standards, rare diseases, metadata

## Abstract

**Background:**

Patient registries are often a helpful first step in estimating the impact and understanding the etiology of rare diseases - both requisites for the development of new diagnostics and therapeutics. The value and utility of patient registries rely on the use of both well-constructed structured research questions and relevant answer sets accompanying them. There are currently no clear standards or specifications for developing registry questions, and there are no banks of existing questions to support registry developers.

**Objective:**

This paper introduces the [Rare Disease] PRISM (Patient Registry Item Specifications and Metadata for Rare Disease) project, a library of standardized questions covering a broad spectrum of rare diseases that can be used to support the development of new registries, including Internet-based registries.

**Methods:**

A convenience sample of questions was identified from well-established (>5 years) natural history studies in various diseases and from several existing registries. Face validity of the questions was determined by review by many experts (both terminology experts at the College of American Pathologists (CAP) and research and informatics experts at the University of South Florida (USF)) for commonality, clarity, and organization. Questions were re-worded slightly, as needed, to make the full semantics of the question clear and to make the questions generalizable to multiple diseases where possible. Questions were indexed with metadata (structured and descriptive information) using a standard metadata framework to record such information as context, format, question asker and responder, and data standards information.

**Results:**

At present, PRISM contains over 2,200 questions, with content of PRISM relevant to virtually all rare diseases. While the inclusion of disease-specific questions for thousands of rare disease organizations seeking to develop registries would present a challenge for traditional standards development organizations, the PRISM library could serve as a platform to liaison between rare disease communities and existing standardized controlled terminologies, item banks, and coding systems.

**Conclusions:**

If widely used, PRISM will enable the re-use of questions across registries, reduce variation in registry data collection, and facilitate a bottom-up standardization of patient registries. Although it was initially developed to fulfill an urgent need in the rare disease community for shared resources, the PRISM library of patient-directed registry questions can be a valuable resource for registries in any disease – whether common or rare.

**Trial Registration:**

N/A

## Introduction

The value and utility of patient registries are largely contingent upon the use of both high-quality research questions and structured and relevant answer sets accompanying them. The interoperability of registries or registry data—including the feeding of registry data into more rigorous clinical studies and regulatory submissions for new agents—depends upon the use of data standards; yet, there is currently no clear specification for developing registry questions nor are there banks of existing questions to support registry developers. The diverse nature of registries, sponsors, and disease-specific data requirements complicate efforts at standardization in registry applications. This paper introduces the *[Rare Disease] PRISM *(Patient Registry Item Specifications and Metadata for Rare Disease) project, a library of standardized questions covering a broad spectrum of rare diseases that can be used to support the development of new registries in all disease areas. If widely used, PRISM will enable the re-use of questions across registries, hence reducing the variation in registry data collection and facilitating a bottom-up standardization of patient registries. Although it was developed to support the rare disease community’s urgent need for shared resources, the PRISM library of patient-directed registry questions can be a valuable resource to registries for all diseases.

### Background

#### Rare Diseases Registries

Often patient registries are a helpful first step in estimating the impact and understanding the etiology of rare diseases—both requisites for the development of new diagnostics and therapeutics. Because of the small numbers of patients affected by rare diseases, these registries present unique challenges related to registry design, enrollment of patients, and data collection [1-5], as well as for analysis and interpretation of registry data [5,6]. In recent years, the U.S. Food and Drug Administration (FDA) and umbrella patient advocacy support organizations (eg, the Genetic Alliance) have publicly encouraged rare disease patient advocacy groups (PAGs) to develop registries as part of a comprehensive strategic research plan. As such, a proliferation of patient registries is underway, and there are currently no dedicated or centralized efforts for developing standards that will reduce the time required to develop new patient registries and facilitate opportunities for shared data.

#### Patient Registry Variation and Standards

There is tremendous variability in the type of data and specific questions that patient registries collect, due in part to the lack of registry-specific data standards and also to the heterogeneity of registries’ purposes and sponsors. Patient registries can be designed for many purposes, including public health surveillance, epidemiologic and longitudinal research, patient education, research recruitment, and population monitoring for the safety of post-marketed drugs and devices. Patient registries can include data reported by patients, researchers, or clinicians. (A characterization of registry types is summarized in Richesson and Vehik [2].) Sponsors and developers of patient registries are varied as well and include governments, academic scientists, and clinical investigators. Often patient registries (and the supporting questions, typically targeted to patients and caregivers rather than physicians) are developed ad hoc by the PAGs themselves, and there is currently no clear specification for standards or banks of existing questions for them to access. Further, the content of these registries (ie, the questions and associated answer sets) may change over time as more becomes known about the disease and its clinical variations, or when new therapeutics and devices become available. There is a tremendous need—especially for the thousands of rare diseases that do not have patient registries—for resources that help registry sponsors and developers to identify well-constructed and meaningful questions.

There is also a clear role for data standards to promote shared efficiencies in registry development and enable opportunities for data sharing. These needs are particularly pronounced for rare diseases, which have sparse resources and significantly fewer—and highly distributed—domain experts and affected patients. An important standards challenge is the fact that there is no central control of patient registries—there is no single funding or regulatory agency that can oversee all the different registry types and implementations. Because registries are developed by many sponsors to address distinct functions, a top-down standards effort would require countless stakeholders and is not feasible. Additionally, there is no central authority to monitor or enforce standards compliance once developed. Given the tremendous need for standards and the scope of data collected across disease-specific registries, and given that there is no incentive or regulatory means to develop standards or enforce compliance, alternatives to complement traditional Standards Development Organizations (SDOs) are needed. Non-traditional strategies for developing and promoting standards can be effective and embraced across various rare diseases if these various research communities perceive them as accessible, useful, helpful, and easy to adopt.

The PRISM project (funded by an American Recovery and Reinvestment Act (ARRA) grant administered through the National Library of Medicine (NIH), NLM Grant Number 1RC1LM010455-01, and supported by the Office of Rare Diseases Research) was developed to provide a useful resource to promote the efficient development of patient registries and standardized quality data collection by supporting the sharing and re-use of existing registry questions and data standards. The fundamental idea behind PRISM is that if registry developers could access questions used by other rare disease registries, they could consider and likely re-use these questions, thereby reducing the variation in questions/data collection across various patient registries, and leading to a *bottom-up *development of standards. In addition, the utility and scalability of PRISM is based on the notion that the PRISM content is accessible and open to any registry sponsor, regardless of prior standards knowledge or experience. The authors are engaged with various standards and informatics organizations and deliberately designed PRISM to facilitate linkage with other standards and research resources as appropriate. The PRISM project, therefore, provides not only a library of questions but a vehicle for the registry developers and rare disease organizations to interact, learn, and develop consensus requirements, which can in turn be directed to various standards development organizations (eg, CDISC [7], HL7 [8], LOINC [9]), research initiatives (eg, caDSR [10] and CSHARE, PROMIS [11,12], PhenX [13]), and national interoperability initiatives emerging from the U.S. DHHS, Office of the National Coordinator [14,15].

As a demonstration project, PRISM explored foundational issues related to the types of questions included in the bank (relative to other standards and question repositories) and the inclusion of metadata that will facilitate their search and retrieval. We describe our methodological approach to developing the PRISM library in the Methods section and present the resulting library structure, features, and composition afterward in the Results section.

## Methods

The first questions identified for PRISM included a convenience sample of questions from well-established (>5 years) natural history studies in various diseases (metabolic [16], vascular [17], developmental disorders [18]) and several existing registries. The questions were obtained largely from the NIH-funded Rare Diseases Clinical Research Network as the authors worked in the data center for the network and had familiarity with the various studies and investigators. These were examined by many experts (both terminology experts at the College of American Pathologists (CAP) and research and informatics experts at the University of South Florida (USF)) for face validity, including commonality, clarity, and organization. Questions were re-worded slightly, as needed, to make the full semantics of the question clear and to make the question generalizable to multiple diseases as possible. For example, a registry data entry item “Genetic test?” would be entered into PRISM as “Have you had a genetic test to confirm your diagnosis?”, representing the intended semantics of the question in a generalizable way, rather than “Have you had a genetic test to confirm your Rett Syndrome diagnosis?”, which was the intent of the question from the source document. In addition, some registry questions were taken from a variety of established rare disease patient registries that authors were acquainted with. To ensure that questions were “stable” and field-tested, authors used well-established content that had been considered final by a PAG after a multi-disciplinary review process and pilot testing. Generic content relative to many or all patient registries was also incorporated, including standard elements from the Rare Diseases Clinical Research Network (RDCRN) Contact Registry (supporting over 200 rare diseases) [19], the OMB and NIH demographics [20], selected questions from the NINDS Common Data Elements [21], and all of the data elements recommended for the Global Registry for Rare Diseases [22], an initiative led by the U.S. Office of Rare Diseases Research. A typology was developed to organize the questions in PRISM and to support internal curation of the library. Questions were indexed by one or more keywords that describe the general content category (eg, demographic, medications, medical history, special histories, etc.). The specific representations of the metadata keywords include common forms (eg, Demographics, Medical History) and form headings (eg, Medication History, Special Education Services) that group related data (by type or by source) for registries and observational studies.

We describe our selected strategy and design features for PRISM in the next section in terms of content, search and retrieval requirements, indexing model and metadata, and strategy for growing the content of the PRISM library. The authors thoroughly explored other standards throughout the design of PRISM and present the relationships and definitions between PRISM and other efforts related to standardized questions and patient reported data. Finally, in the discussion section, we describe immediate future directions for PRISM, including requirements for sharing the library, interface design, and future plans for maintenance and governance of PRISM.

## Results

### Content

At present, PRISM contains over 2,200 questions. A sample of 224 questions and selected metadata is presented in Appendix 1. Many questions (especially the most general, such as “List current medications you are taking” and “List any other major diseases you have had”) are relevant to virtually all rare diseases, and others are relevant to a great many rare diseases (eg, “Do you require an assistive device for walking?”, or “Was your child born full-term?”). However, the majority of PRISM questions—such as “Does your child hoard food?”, “How many times per week does child pick at own skin until it bleeds?”, “Does your child need TV to fall asleep?”, and “Approximately how many bone fractures have you had in your lifetime?”—are relevant only to particular diseases, and often the valid answer sets associated with each question are disease-specific. For example, valid answers to the question “What age was your child’s first bone fracture?” would include prenatal ages in a patient registry targeted to Osteogenesis Imperfecta, thereby requiring different units and ranges of expected values. The obvious challenge that thousands of such disease-specific questions (relevant to the thousands of rare diseases seeking to develop registries) will present for traditional standards development organizations is what drove the design decision for the scope of PRISM. The scope of PRISM, therefore, deliberately includes a range of disease-specific content, along with narrative definitions and metadata for indexing and source preservation. The metadata was selected to facilitate linkages between rare disease communities and existing SDOs and controlled terminologies, as described in the following sections.

### Related Efforts

To prevent overlapping with other standards efforts related to the collection of clinical data, a deliberate search for relevant standardization efforts was undertaken by the authors. This search of existing standards and informatics and library resources revealed several related and potentially relevant efforts, and informed the design of PRISM to leverage related efforts. As described in the introduction, the focus of PRISM is on *patient-reported *questions that are not a part of standardized, validated patient assessment instruments. Given that focus, several organizations are engaged in various attempts to inventory and codify standardized assessment instruments and items, specifically Clinical LOINC [23], PROMIS, the caDSR and NINDS Common Data Elements [24], and to some extent SNOMED CT. Because PRISM was developed with the intent to leverage and coordinate existing standards as much as possible, the PRISM scope was clearly defined at the onset to prevent any overlap. However, some areas of potential overlap with other initiatives exist. Table 1 and Table 2 describe the potential relationship of PRISM to other initiatives that are also developing repositories of questions or data elements.

**Table 1:** Relationship of PRISM to Related Standards Efforts and Resources (LOINC, caDSR, and PhenX).

Initiative^a^	Primary Sponsor	Objective	Scope of standard	Proposed relationship with PRISM
Clinical LOINC http://www.webcitation.org/6BJJ3JZIm	NLM	Messaging and interoperability of clinical information. Specifically, the LOINC database provides a set of universal names and ID codes for identifying laboratory and clinical test results.	Health care (primarily) and research	Patient assessment scales are not generally included in PRISM. PRISM documentation directs users to LOINC for this content. [Note: Clinical LOINC does not contain every assessment scale ever published. Registry developers need to search Clinical LOINC or RELMA.] LOINC is interested in supporting electronic health record (EHR) data standards. The rare disease community should leverage LOINC to support transfer of EHR data to registries. PRISM could coordinate this. The majority of PRISM content is variable and might not be messaged or collected in EHRs. But, some PRISM items can be submitted to LOINC if appropriate. PRISM will filter and act as a feeder of selected content into LOINC; PRISM definitions and metadata will aid in this.
caDSR (CSHARE) http://www.webcitation.org/6BJJ5fd1Y	NCI	Research data elements. “caDSR is a database and a set of APIs and tools to create, edit, control, deploy, and find common data elements (CDEs) …for use in software development.” (CSHARE is expected to emerge as an expanded pan-disease version of the caDSR but is not yet available.)	Data elements for collection in clinical research studies. NCI’s vocab services and metadata repositories do support diseases other than cancer, and have standard data elements from FDA and NIH institutes and centers.	caDSR content and tools are targeted to clinical researchers. Much of caDSR content could be relevant to PRISM and rare disease registry developers, but is not complete nor easily searchable by rare disease users. PRISM includes a focus on registries and rare diseases and a community forum for rare disease registry standards. PRISM maintains a link to source for all content. Users can see explicit links to caDSR if that is the source. PRISM questions could be imported into caDSR for use in clinical research projects. (The PRISM metadata has enough detail to support their transfer if caDSR curators want.)
PhenX http://www.webcitation.org/6BJJ8xNzU	National Human Genome Research Institute (NHGRI)	To provide investigators with high-quality, relatively low-burden measures for inclusion in genome-wide association studies (GWAS) and other large-scale research efforts.	Data elements used in new research data collection, or used/queried from various electronic health records. Content focuses on common diseases but is growing.	Much of PRISM content is very disease specific, often idiosyncharic, and not included in PhenX. PhenX is consulted as a resource for generic questions, which were incorporated into PRISM when authors thought appropriate (underlying PRISM database includes PhenX code in these cases). Where content overlap exists, PRISM points users to PhenX measures. PhenX is aimed at researchers, but user interface is intuitive and easily accessible to PRISM users.

^a ^These initiatives are not specific to patient registries or rare diseases.

**Table 2:** Relationship of PRISM to Related Standards Efforts and Resources (PROMIS, SNOMED CT, RxNorm).

Initiative^a^	Primary Sponsor	Objective	Scope of Standard	Proposed relationship with PRISM
PROMIS (Patient Reported Outcomes Measurement Information System) http://www.webcitation.org/6BJIvffJ8	NIH	A system of highly reliable, precise measures of patient–reported health status for physical, mental, and social well-being.	Functional and quality-of-life assessment questions. Validated measures only; focus on psychosocial constructs across domains, not only specific diseases.	PRISM explicitly avoids content that is validated and intended to be used for measurement. Where there are common questions, PROMIS and PRISM will cross-reference each other. PRISM directs users to PROMIS and describes its potential application in patient registries.
SNOMED CT (SCT) http://www.webcitation.org/6BJIyeJ5C	Int’l. standards development organisation (IHTSDO) [Supported by dues from member nations] US residents may use SNOMED CT free of charge, supported by NLM.	Provides a consistent way to index, store, retrieve, and aggregate clinical data across specialties and sites of care.	Comprehen-sive clinical terminology covering nursing and medical diagnoses, signs and symptoms, functional status, interventions, procedures, and outcomes.	SCT is used for indexing in PRISM. Each PRISM question associated with one or more codes that best represent the important content of the PRISM QAS. The most specific SCT code is used, with the understanding that only some PRISM questions get very precise representation in SCT. Similarly, multiple SCT codes can be used to index the clinical content of multi-concept questions. [In 2006, the SCT nursing working group (prior to the IHTSDO) developed a model for coding assessment scales to SCT, which was approved by the SCT International Editorial Board. So, SCT should be added to the “specifically” list. The Observable Entity-Answer approach to coding PRISM follows the SCT model for coding assessments.]
RxNorm	NLM	RxNorm contains the names of prescription and many non-prescription formulations in the US; aims to support electronic exchange of medication information and clinical decision support related to CPOE in health care contexts.	Standardized nomenclature for clinical drugs and drug delivery devices (mostly in US); gives normalized names for clinical drugs and links its names to many drug vocabularies used in pharmacy management and drug interaction software.	RxNorm does not represent data elements but is a nomenclature for clinical drugs. Many registries ask questions about specific medications. RxNorm is used for indexing questions about medications in PRISM. Each medication-related question in PRISM is associated with RxNorm codes that best represent the named drug – either by clinical drug name, (generic) ingredient, or packaged products.

^a ^These initiatives are not specific to patient registries or rare diseases.

Some data elements, like height and weight are in clinical data element repositories such as Clinical LOINC and research data element registries like caDSR. To enable “one-stop shopping” for registry developers, these items are also in PRISM, with the idea that future PRISM interfaces can identify linkages to these other standards where and when appropriate. These linkages can inform PRISM users that they indeed are using items from another designated standard and can also inform PRISM curators to ensure that they do not create or support future variations in that item.

In the interest of rapidly assembling content relevant to patient registries for any and all rare diseases, the initial PRISM strategy has been to accept virtually all questions, with the idea that either patient communities or curators might later filter, rate, or rank them for PRISM users. Despite limiting the inclusion of questions to those from well-established registries, some questions used in rare disease registries were poorly constructed or not as clear as they could be. Because PRISM is motivated to address the registry question needs for a spectrum of registry designs and diseases, it does contain registry data elements (in actual use) that might not be ideally constructed or might actually conflict with another value set. Regardless, the liberal acceptance policy of PRISM increases the breadth and volume of questions and ultimately increases the value of PRISM as a central resource for questions (which should be supplemented by advice on selection and use). We hope that others use PRISM as a resource for standards development or build applications that can facilitate the ranking or endorsement of certain PRISM questions over others in specific diseases or contexts.

### Search and Retrieval Requirements

As mentioned in the previous section, the growth of the library brought with it challenges for curation and use. Internally, a process was developed to add new content without duplicating questions and ensure that related questions or variants could be indexed for effective retrieval and comparison. We operated under the assumption that PRISM needed to be useful to provide value. We explored user roles and searching techniques to determine the best method for indexing question and answer sets (QAS). Our indexing scheme (including the use of controlled terminology) is described later. A key strategy for identifying the search and retrieval requirements was employing use cases.

The PRISM team developed narrative *use cases *(for question searching, re-use, and new registry development) that have informed our indexing approaches. For our indexing strategy, PRISM also developed use cases of retrieval requirements as a guide of needed functionality and detail, and leveraged existing metadata and controlled vocabularies. The use cases describe the development of new registries using PRISM as a resource for the questions. Specifically, the use cases describe the development of a prospective vasculitis and pregnancy registry—including the re-use of PRISM questions and the submission of new questions to PRISM—and the subsequent development of another registry using these same pregnancy-related questions. Finally, the use cases articulate a demonstration and initial proof of the interoperability of data collected from different registries and data sources. These functional use cases ultimately will also support the evaluation of PRISM. The use cases were developed by the authors and not widely vetted in the rare disease community. They can be found on PRISM website [25].

### Indexing Model and Metadata

A critical and largely unaddressed problem for registries (and clinical research data collection in general) is the need for patient registry questions and answers to be indexed in such a way that they can be retrieved for re-use, for example, to support rapid development of another related rare disease registry. In essence, indexing is the practice of applying metadata (structured and descriptive information) to items in a database for efficient and accurate retrieval [26]. An important measure of indexing is specificity, which refers to the detail or precision of the indexing process and its depth [26(p161)]. Achieving the appropriate level of specificity in encoding the question and answer sets was a continuous challenge. Our approach was guided by the ideal situation where the pairs would be encoded to convey the semantic meaning of the clinical concepts and at the same time allow efficient collocation. The specificity and overall indexing approach informed the detailed use cases discussed earlier. The indexing scheme also addresses semantic ambiguities inherent in research questions and answer sets, and includes metadata for the following constructs:

1. Context (type of study, disease or treatment of interest, etc.)

2. Format of questions and location of semantics

3. Who is asking the question (patient, relative, doctor)?

4. Audience or person being asked the question (patient, family member or caregiver)

5. Relevant data standards for specific answer sets

### Metadata and Controlled Vocabularies

Metadata are used to describe information resource-type features of questions, such as terms and attributes, and controlled vocabularies represent the actual content. Both are important for retrieval and, ultimately, interoperability. PRISM uses Dublin Core (DC) as a metadata framework for indexing PRISM QAS, and within DC metadata uses controlled terminology to reflect the semantics for each question. Our approach is described in detail in [27]. Terminology control, when implemented correctly and consistently, can dramatically improve the quality of search results in most contexts. SNOMED CT is an ONC recommended standard for many aspects of the electronic medical record, and previous research has indicated that SNOMED CT is also well suited for clinical concepts in research [28].

The use of Dublin Core metadata to annotate various QAS in PRISM offers a way to employ the most appropriate controlled vocabulary(s) for the content, while preserving retrievability. In addition to selected Dublin Core metadata elements and controlled terminologies, other decisions were made to ensure that these elements and vocabularies were used appropriately and consistently. Specifically, assuring each QAS is usable, reproducible, and understandable on its own merit. For example, a form that addresses gynecological issues may include a QAS addressing menstrual symptoms, such as “Do you experience cramps?” From the context of the form, this can refer only to menstrual cramps and may be coded with or without the “menstrual semantics”. However, when a later user searches the registry for library questions about abdominal, leg or other body site cramps, this question may be inappropriately selected. The QAS metadata (including the embedded SNOMED CT codes and the narrative definition of the question that PRISM includes) can be used to easily disambiguate this term.

Under the leadership of terminology experts from the College of American Pathologists, guidelines for using SNOMED CT were developed related to post-coordination, selection of hierarchies, and level of specificity. Guidelines for the use of SCT within the PRISM Library data model were developed collaboratively by the PRISM team and included the following decisions:

1. Assigning codes for entire question and answer set groups vs. discrete codes for questions and answers

2. An approach to take the (semantically) closest available SNOMED CT concept rather than creating a new one

3. Consequently, we considered but rejected the idea to create a SNOMED extension (“Ref Set”) mechanism

4. Versioning and change protocols were developed between USF and CAP partners

### Procedures for Adding New Items / Growing PRISM content

As described in the previous section, PRISM has developed a useful, consistent, and standards-compliant solution for the encoding of questions in PRISM. Ultimately, the metadata model and indexing strategy will be tested as the content of PRISM grows. Implementation plans should ensure that as the size of the PRISM library grows, duplicate questions are not inadvertently added. (Anecdotally, this is an issue with other question and metadata repositories, owing to the fact that a complete search of existing content must be undertaken before new content is added, and this search is both time-consuming and generally not incentivized.) For PRISM to remain a useful resource to rare disease registry developers, and for PRISM to support the goal to reduce question variation across rare disease registries, the library should not contain obvious duplicates and the library indexing model should be sufficient for users to search *and retrieve *relevant registry questions from PRISM before they re-create their own variation. As new content is introduced to the PRISM outreach team at USF, they first determine if the content is in scope of PRISM (ie, is not purview of other standards initiatives shown in Table 1 and Table 2). Then, as questions are identified for possible addition into the library, library professionals perform multiple and semantically enhanced queries to compare to QAS already in the database. Through collaborations with rare disease stakeholders who are part of the PRISM scientific review committee, they are then edited for structure and clarity, and definitions are provided. After the questions have been edited, semantic and administrative metadata are applied (including controlled terminology) before integrating them into the library’s database.

## Discussion

### Limited Scope

**Figure 1:**
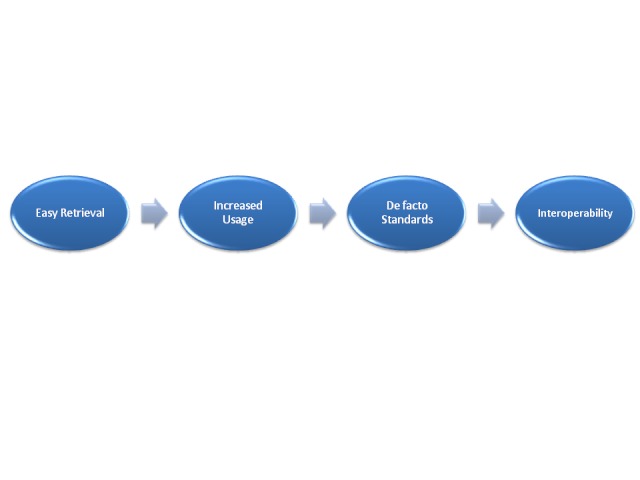
Theory of PRISM design to interoperability.

One of the biggest challenges of the PRISM project has been to keep the scope reasonable and practical. Since the targeted audience for PRISM is researchers and registry developers (including PAGs with non-research backgrounds), we took a minimalist approach to coding with the goal of easy retrieval. It is our expectation that easy retrieval will drive increased usage, which will cultivate de facto standards, and those standards will ultimately support interoperability (see Figure 1).

We recognize that interoperability between registry questions and other data (eg, EHR data) would require more sophisticated coding with SNOMED CT and other data standards. Given the short duration of this project and the desire for maximum retrievability, we determined that this level of coding would be out of the scope for PRISM at this time.

It is not clear at this point how large a corpus of sharable items that there is among disparate and different rare diseases. Likewise, it is still unclear whether items for rare diseases are likely to be similar to items for more common diseases, and if so, whether there would be value in finding a way to include and reuse those items via PRISM as well. The future use and evaluation of PRISM content by multiple disease representatives will yield information on the reusability of the questions in PRISM within and across rare and common diseases, as well as provide practical examples of cross-disease standards and determination of standards gaps. To understand the reusability and generalizability of PRISM questions, additional work needs to explore the validity and reproducibility of the categorization and indexing of questions.

### The Future

PRISM fills a void for the rare diseases research and registry development communities. The PRISM library resource can support standardized data collection in patient registries by reducing unnecessary variation. PRISM is free and available to search through the project website [25]. Authors are also in the process of making the PRISM library metadata model and content accessible through the National Center for Biomedical Ontology [29] Bioportal. While the library content is available, we encourage innovators and developers to build tools that integrate PRISM content into registry development efforts. It is possible that the PRISM library resource could be leveraged in large research data collection tools such as the NIH-funded REDCap project.

Authors are hopeful for future funding that will allow PRISM content to grow to meet the needs of the thousands of rare diseases registry applications and to allow computer mediated methods for adding and presenting content. The notion of using a distributed community of registry developers to curate this resource by commenting on and ranking items—as with the demonstration of caDSR content that is described in [30]—is very appealing. Such projects would require extensive marketing and publicity of PRISM to a comprehensive group of rare disease registry stakeholders and researchers in order to bolster the extensive use of PRISM that would be required to effectively demonstrate a bottom-up community driven development of standards. An increased usage and future growth of PRISM will in turn require formal governance for PRISM and official policies related to content and a submissions and update processes.

The PRISM developers, with the cooperation and support of the National Organization for Rare Disorders (NORD), are working to make the PRISM resource available and useful to registry developers representing all rare diseases and all countries. Currently, several rare disease patient advocacy organizations are participating in focus groups and expert interviews to inform the development of best interfaces and retrieval strategies to ensure that PRISM is a useful and accessible community-driven resource. In addition, we are developing international collaborations to explore the translation of items to support global rare disease research. Our overarching goal is that—given the sheer number of rare diseases, the variety of registry designs, and the number of languages that might need to be addressed—the PRISM leadership seizes and implements standards opportunities without burdening resource-strapped rare disease communities.

### Summary

The lack of a clear set of standards and specifications for data collection using patient registries represents a significant data standards gap in an explosively growing application area—important to both drug development and patient-directed health communities. Standardization of patient registries can enable the interoperability of health and research data, as registries should be able to receive data from health care system or transmit data into various clinical research or pharmacovigilance applications. PRISM can be used to facilitate interoperability of existing and newly developed registries and to ensure that moving forward, registries use standard sets of questions. Without the use of such a resource, the proliferation of patient registries and variation of data collection questions will be inevitable. This central resource, the PRISM library, will support a bottom-up and incremental standards promulgation. By using a standard set of metadata elements and SNOMED CT to facilitate the retrieval and re-use of existing questions and standards, PRISM will reduce variation in the rare diseases registry community and assist registry implementers to produce high quality registries much more efficiently than ever before. Once variation in patient registries is reduced (ie, “standards” emerge), then issues related to harmonizing, mapping, and relating to the different standards communities for health care (eg, HL7) and research (eg, CDISC) can be addressed in an efficient manner. In this approach, the standardization of patient registry questions can serve to improve efficiencies, collaboration, and resource sharing across the entire drug development process.

Information regarding the development of PRISM and access can be found on the PRISM website [24] .

## Multimedia Appendix 1

Sample questions and selected metadata.

## Abbreviations

caDSR: Cancer Data Standards Registry and Repository
CAP: College of American Pathologists SNOMED Terminology Solutions
CDISC: Clinical Data Interchange Standards Consortium
CSHARE: CDISC Shared Health And Clinical Research Electronic Library (http://www.cdisc.org/cdisc-share)
DC: Dublin Core Metadata Initiative
HL7: Health Level Seven
LOINC: Logical Observation Identifiers Names and Codes
NINDS: National Institute of Neuromuscular Disorders and Stroke
NLM: National Library of Medicine
NORD: National Organization for Rare Disorders
OMB: The White House Office of Management and Budget
ONC: Office of National Coordinator for Health Information Technology
ORDR: Office of Rare Diseases Research
PAG: Patient Advocacy Group
PhenX: Consensus measures for Phenotypes and eXposures
PRISM: Patient Registry Item Specification and Metadata
PROMIS: Patient Reported Outcomes Measurement Information System
QAS: Question and Answer Set
RDCRN: Rare Disease Clinical Research Network (http://www.rarediseasesnetwork.org)
SCT: SNOMED CT
SDO: Standards Development Organization
SNOMED CT: Systematized Nomenclature of Medicine–Clinical Terms
USF: University of South Florida
