# Supplementary material for: Standardization of Questions in Rare Disease Registries: The PRISM Library Project
Source: Interact J Med Res. 2012 Oct 10;1(2):e10. doi: 10.2196/ijmr.2107 (PMC3626121; doi:10.2196/ijmr.2107)
Supplement: Supplementary file 1 [file ijmr_v1i2e10_app1.pdf]

A Sample of PRISM Questions and Selected Metadata

Title	Permissible Values (SNOMED Codes)	Units	Question Type	Format	Category	Audience	Question Date	Coverage	QuestionDescription	Mesh	Author	Publisher	ResourceType	ResourceId	Language	References
Did you ever have sinusitis?	☐ Yes<br>☐ Unknown<br>☐ No		question	single-select	Medical history	patient	02/02/2011	Yes/no indicator to ask if patient ever had sinusitis (Inflammation of the nasal mucosa in one or more of the paranasal sinuses)	sinusitis indicator	sinusitis	Primary Immune Deficiency Treatment Consortium	PRISM	question	CAP to provide	en	
Have you ever been diagnosed with myositis? [Proximal muscle aching/weakness,	☐ No<br>☐ Yes		scale	single-select	Disease specific medical history	patient	02/16/2011	Yes/no indicator to ask if patient was ever diagnsed with myositis, an Inflammation of a	myositis indicator	myositis	Bombardier, C; Gladman, DD; Urowitz, MB; Caron, D, Chang	PRISM	scale	CAP to assign	en	Bombardier, C; Gladman, DD; Urowitz, MB; Caron, D; Chang DH and the Committee on Prognosis studies in SLE (1992).

associated with elevated creatine phsophokinase/adolase or electromyogram changes or a biopsy showing myositis]								muscle or muscle tissue.			DH and the Committee on Prognosis Studies in SLE					Derivation of the SLEDAI: A disease activity index for lupus patients. Arthritis Rheum 35, 630-40.
How often does sleepiness interfere with your child's school/university?	☐ Never<br>☐ Daily<br>☐ Once a week<br>☐ Once a month		question	single-select	Disease symptoms	patient	11/02/2010	Frequency, as reported by parent, that sleepiness	Text term to signify a person's self-report of how often sleepiness interferes with child's school/university	Disorders of Excessive Somnolence	Angelman, Rett, Prader-Willi Syndromes Consortium	PRISM	question	CAP to provide	en	
Has your child ever been given any of the following diagnoses? (Indicate all that apply)	☐ Attention Deficit Disorder<br>☐ Attention Deficit Hyperactivity Disorder<br>☐ Pervasive Developmental Disorder<br>☐ Other<br>☐ Oppositional		question	multi-select	Diagnosis	patient	01/12/2011	Enumerated list to identify child's psychological diagnoses	behavioral diagnosis indicator	diagnosis	Angelman, Rett, Prader-Willi Consortium	PRISM	question	CAP to provide	en	

	Defiant Disorder<br>□ Autism<br>□ Obsessive Compulsive Disorder<br>□ Conduct Disorder															
How often does your child have trouble sleeping away from home (visiting relatives, vacation, etc.)?	□ Rarely: 0 to 1 time during week<br>□ Usually: 5 or more times a week<br>□ Sometimes: 2 to 4 times during week		scale	single select	Child behavior	patient	11/08/2010	Frequency (number of times per week) that a child	Text term to signify person's self report of how often child has trouble sleeping away from home (visiting relatives, vacation, etc.)	sleep disorders	Judith Owens, M.D., MPH	PRISM	scale	CAP to provide	en	Judith Owens, MD, MPH. Child's Sleep Habits Questionnaire (CSHQ: Preschool and School-Aged abbreviated version). Version 2.0 http://www.kidzzzsleep.org
How often does sleepiness interfere with your school/university?	□ Never<br>□ Daily<br>□ Once a week<br>□ Once a month		question	single-select	Disease symptoms	patient	11/02/2010	Frequency that sleepiness interferes with patient'	Text term to signify person's self-report of how often sleepiness interferes with school/university	Disorders of Excessive Somnolence	Angelman, Rett, Prader-Willi Syndromes Consortium	PRISM	question	CAP to provide	en	
How would you describe the severity of your	□ More severe since		question	single-select	Disease symptoms	patient	02/01/2011	Enumerated list to indicate severity of	child severity of seizures indicator	seizure disorders	Angelman, Rett, Prader-Willi	PRISM	question	CAP to provide	en	

child's seizures?	study started<br>☐ Not applicable (initial study visit)<br>☐ No change since study started<br>☐ Less severe since study started							child's seizures			Consortium					
How would you describe your child's motor ability?	☐ Better since study started<br>☐ Not applicable (initial study visit)<br>☐ No change since study started<br>☐ Worse since study started		question	single-select	Disease symptoms	patient	02/01/2011	Enumerated list to indicate level of child's motor ability (Marked impairments in the development of motor coordination such that the impairment interferes with activities of daily living)	child motor ability indicator	motor skill disorders	Angelman, Rett, Prader-Willi Consortium	PRISM	question	CAP to provide	en	
How would you describe your child's	☐ Sits stably with no		question	single-select	Disease symptoms	patient	02/01/2011	Enumerated list to indicate	child trembling/shaking	postural balance	Angelman, Rett, Prader-	PRISM	question	CAP to prov	en	

balance/coordination for sitting?	support<br>☐ Sits wobbly<br>☐ Unable to sit without falling<br>☐ Sits, but needs to be supported on 2 to 3 sides<br>☐ Sits, but needs back support							child's balance for sitting (a posture in which an ideal body mass distribution is achieved. Postural balance provides the body carriage stability and conditions for normal functions in stationary position or in movement, such	indicator		Willi Consortium			ide		
How would you rate your child's use of nonverbal communication?	☐ Better since study started<br>☐ Not applicable (first study visit)<br>☐ No change since study started<br>☐ Worse since		question	single-select	Child development	patient	02/01/2011	Enumerated list to indicate how parent rates child's nonverbal communication	child non-verbal communication indicator	non-verbal communication	Angelman, Rett, Prader-Willi Consortium	PRISM	question	CAP to provide	en	

	study started															
Does your child have any new behavioral skills? (ex. Turn-taking, putting things in a box, sharing, using toys or tools correctly, pretneding activities, social interactivity)	☐ Yes (specify)<br>☐ Not applicable (first study visit)<br>☐ No		question	single-select	Child development	patient	02/01/2011	Yes/no indicator to ask if child has any new behavioral skills	child new behavioral skills indicator	child behavior	Angelman, Rett, Prader-Willi Consortium	PRISM	question	CAP to provide	en	
Has your child lost any behavioral skills?	☐ Yes (specify)<br>☐ Not applicable (first study visit)<br>☐ No		question	single-select	Disease symptoms	patient	02/01/2011	Yes/no indicator to ask if child lost any behavioral skills	child lost behavioral skills indicator	child behavior	Angelman, Rett, Prader-Willi Consortium	PRISM	question	CAP to provide	en	
Do you use a C-PAP (continuous positive airway pressure) device?	☐ Yes<br>☐ Unknown<br>☐ No		question	single-select	Therapies	patient	01/24/2010	Yes/no indicator to ask whether a patient uses a c-pap device (device which maintains airway pressure above	c-pap device indicator	continuous positive airway pressure	Osteogenesis Imperfecta Foundation	PRISM	question		en	

								atmospheric pressure throughout the respiratory cycle by pressurization of the ventilatory circuit)								
What did your child weight at most recent visit to the doctor?	ounces<br>pounds		question	single-select	Anthropometry	patient	02/01/2011	Text indicator to specify child's weight at most recent doctor visit	child weight indicator	body weight	Angelman, Rett, Prader-Willi Consortium	PRISM	question	CAP to provide	en	
How would you describe your child's oral sensitivity?	None<br>Occasionally reacts to new textures or temperatures<br>Rejects touchng of lips and tongue<br>Accepts touching lips or tongue but does not allow food in		question	single-select	Disease symptoms	patient	02/01/2011	Enumerated list to indicate child's oral sensitivity	child oral sensitivity indicator	oral manifestations	Angelman, Rett, Prader-Willi Consortium	PRISM	question	CAP to provide	en	

	mouth<br>☐ Allows food in mouth, but reacts strongly to new textures or temperatures															
How severe has your mouth pain been at its worst in the last week?	☐ 0 (Not Present)<br>☐ 1<br>☐ 2<br>☐ 3<br>☐ 4<br>☐ 10 (As bad as you can imagine)<br>☐ 6<br>☐ 7<br>☐ 8<br>☐ 9<br>☐ 5		question	single-select	disease-specific symptoms	patient	02/21/2011	Enumerated list to indicate severity of mouth pain (An unpleasant sensation induced by noxious stimuli which are detected by nerve endings of nociceptive neurons)	mouth pain	pain; mouth	Immune Mediated Disorders After Allogenic HCT	PRISM	question	CAP to assign	en	
How severe has your mouth sensitivity been at its worst in the last week?	☐ 0 (Not Present)<br>☐ 1<br>☐ 2<br>☐ 3<br>☐ 4<br>☐ 10 (As bad as you can imagine)		question	single-select	disease-specific symptoms	patient	02/21/2011	Enumerated list to indicate severity of mouth sensitivity (an unpleasant sensation induced by	mouth sensitivity	pain; mouth	Immune Mediated Disorders After Allogenic HCT	PRISM	question	CAP to assign	en	

	☐ 6<br>☐ 7<br>☐ 8<br>☐ 9<br>☐ 5							noxious stimuli which are detected by nerve endings of nociceptive neurons)								
Does your child have a history of sinusitis [inflammation or infections of the sinuses (hollow air spaces within the bones surrounding the nose)]?	☐ yes<br>☐ unknown<br>☐ no		question	single select	Medical history	patient	12/10/2010	yes / no indicator ask whether a patient has been diagnosed with sinusitis (Inflammation of the nasal mucosa in one or more of the paranasal sinuses)	sinusitis present	sinusitis	Genetic Disorders of Mucociliary Clearance Research Consortium	RDCRN	question	URI	en	
Has your child been diagnosed with situs inversus (organs in the chest and abdomen are opposite to their normal positions)?	☐ yes<br>☐ unknown<br>☐ no		question	single select	Medical history	patient	12/10/2010	yes/no indicator to determine whether patient has been diagnosed with situs inversus (organs in the chest and abdomen are opposite to their normal positions)	diagnosis of situs inversus	Situs inversus	Genetic Disorders of Mucociliary Clearance Research Consortium	RDCRN	question	URI	en	

A Sample of PRISM Questions and Selected Metadata

Has your child been diagnosed with situs solitus?	☐ yes<br>☐ unknown<br>☐ no		question	single select	Medical history	patient	12/10/2010	yes/ no indicator of normal position of organs in thorax and abdomen	diagnosis of situs solitus	situs solitus	Genetic Disorders of Mucociliary Clearance Research Consortium	RDCRN	question	URI	en	
Has your child been diagnosed with abdominal situs inversus (organs in the abdomen are on the opposite side of their normal position)?	☐ yes<br>☐ unknown<br>☐ no		question	single select	Medical history	patient	12/10/2010	yes / no indicator to ask if patient has been diagnosed with abdominal situs inversus (organs in the abdomen are on the opposite side of their normal position)	diagnosis of abdominal situs inversus	abdominal situs inversus	Genetic Disorders of Mucociliary Clearance Research Consortium	RDCRN	question	URI	en	
How many episodes of sinusitis does your child have per year?	☐ 0<br>☐ greater than 10<br>☐ 6 to 10<br>☐ 1 to 5		question	single select	Medical history	patient	12/10/2010	Number of episodes per year of inflammation of the nasal mucosa in one or more of the paranasal sinuses.	number of sinusitis episodes yearly	sinusitis	Genetic Disorders of Mucociliary Clearance Research Consortium	RDCRN	question	URI	en	

A Sample of PRISM Questions and Selected Metadata

What were the results of your child's abdominal ultrasound?	normal<br>abdominal situs inversus<br>polysplenia<br>asplenia<br>central liver		question	single select	Other tests	patient	12/10/2010	result of abdominal ultrasound	abdominal ultrasound findings	ultrasonography	Genetic Disorders of Mucociliary Clearance Research Consortium	RDCRN	question	URI	en	
Did your child have the University of North Carolina Molecular Genetics Laboratory primary ciliary dyskinesia test?	no<br>yes		question	single select	medical history, pulmonary disorders, disorders of mucociliary clearance, cystic fibrosis, primary ciliary dyskinesia, pulmonary disorders, disorders of mucociliary clearance, cystic	patient	12/10/2010	Yes / no indicator to ask whether a patient has had the University of North Carolina Molecular Genetics Lab primary ciliary dyskinesia genetic test	if PCD genetic mutation analysis performed, UNC?	genetic testing	Genetic Disorders of Mucociliary Clearance Research Consortium	RDCRN	question	URI	en	

					fibrosis, primary ciliary dyskinesia											
If your father's father ever received a second diagnosis of breast cancer, was it in the same breast as the first or the opposite?	☐ Opposite breast<br>☐ Same breast		question	single select	Family history	patient	01/06/2011	Text indicator to state whether patient's paternal grandfather's second diagnosis of breast cancer was in the same or opposite breast	Paternal grandfather second diagnosis of cancer in same or opposite breast indicator	breast neoplasms; diagnosis;	Rare Cancer Genetics Registry	PRISM	question	CAP to provide	en	
Has your child ever been diagnosed as overweight?	☐ Yes<br>☐ Unknown<br>☐ No		question	single-select	child history	patient	01/04/2011	Yes/no indicator to ask whether an individual has been diagnosed as overweight (body weight that is above certain standard of acceptable or desirable weight. In the scale of body mass index, overweight is defined as	overweight indicator	overweight	Angelman, Rett, Prader-Willi Syndromes Consortium	PRISM	question	CAP to provide	en	

								having a BMI of 25.0-29.9 kg/m2.)								
Was your child overweight (as determined by your pediatrician) before age 1 year?	☐ Yes<br>☐ Unknown<br>☐ No		question	single-select	child history	patient	01/04/2011	Yes/no indicator to ask whether an child under the age of one year old was been diagnosed as overweight (body weight that is above certain standard of acceptable or desirable weight. In the scale of body mass index, overweight is defined as having a BMI o	overweight indicator	overweight	Angelman, Rett, Prader-Willi Syndromes Consortium	PRISM	question	CAP to provide	en	
Your child's weight at the time s/he was considered to be overweight	☐ Numeric Value	kilograms	question	single-select	child history; anthropometry	patient	01/04/2011	Body weight (in kilograms) at the time of diagnosis of overweight (body weight that is above certain standard of	overweight measurement, weight	body weight	Angelman, Rett, Prader-Willi Syndromes Consortium	PRISM	question	CAP to provide	en	

								acceptable or desirable weight. In the scale of body mass index, overweight is defined as having a BMI of 25.0-29.9 kg/m2), as reported by the								
At what age was your child first able to sit without assistance?	☐ Numeric value	Years or months	question	single-select	child history	patient	01/04/2011	Age at which a child is able to sit up without assistance as reported by the parent	sitting unassisted age	child development	Angelman, Rett, Prader-Willi Syndromes Consortium	PRISM	question	CAP to provide	en	
Through which route (the various ways of administering a medication to a site in a patient from where the chemical is absorbed into the blood and delivered to the target tissue) is the	☐ orally (taken by mouth)<br>☐ intravenously (administered through the veins)<br>☐ sublingually (under the tongue)<br>☐ topically		question	single-select	Concomitant Medications	patient	03/01/2011	Enumerated list text codes and names to represent the access route through which the concomitant medication administered to the patient. Routes are the various ways of	administration route of concomitant medication name	Drug Administration Routes	Rare Diseases Clinical Research Network	PRISM	question		en	

concomitant medication administered to you?	(applied to the skin)<br>☐ Unknown<br>☐ intramuscularly (administered into the muscles)<br>☐ rectally (administered through the rectum)<br>☐ intrathecally (administered into the spinal cord)<br>☐ Other<br>☐ subcutaneously (administered through the skin into the adipose tissue)							administering a drug or other chemical to a site in a patient or animal from where the								
Did your mother's mother ever receive a second	☐ Yes<br>☐ Unknown<br>☐ No		question	single select	Family history	patient	01/06/2011	Yes/no indicator to ask whether patient's maternal	Maternal grandmother's second diagnosis of breast	breast neoplasms; diagnosis;	Rare Cancer Genetics Registry	PRISM	question	CAP to provide	en	

diagnosis of breast cancer?								grandmother ever received a second diagnosis of breast cancer	cancer indicator							
If your mother's mother ever received a second diagnosis of breast cancer, was it in the same breast as the first or the opposite?	☐ Opposite breast<br>☐ Same breast		question	single select	Family history	patient	01/06/2011	Text indicator to state whether patient's maternal grandfmother's second diagnosis of breast cancer was in the same or opposite breast	Site of second diagnosis of maternal grandmother's breast cancer indicator	breast neoplasms; diagnosis;	Rare Cancer Genetics Registry	PRISM	question	CAP to provide	en	
Have you ever had weakness?	☐ Yes<br>☐ Unknown<br>☐ No		question	single-select	Medical history	patient	01/25/2011	Yes/no indicator to ask if patient ever had weakness	weakness indicator	muscle weakness	Bone Marrow Failure Consortium	PRISM	question	CAP to provide	en	
Have any of your family members ever tested positive for BRCA1 (linked to breast and ovarian cancer)?	☐ Yes<br>☐ Unknown<br>☐ No		question	single select	Genetic testing	patient	01/06/2011	Yes/no indicator to ask whether any members of patient's family ever tested positive for BRCA1 (either of two tumor	BRCA1 gene indicator	genes, BRCA1	Finkelstein Rare Cancers Registry Survey	PRISM	question	CAP to provide	en	

								supressor genes that in mutated form tend to be associated with an increased risk of certain cancers and especially breast and ovarian canc								
Have any of your family members ever tested positive for BRCA2 (linked to breast, ovarian, pancreatic, and other cancers)?	☐ Yes<br>☐ Unknown<br>☐ No		question	single select	Genetic testing	patient	01/06/2011	Yes/no indicator to ask whether any members of patient's family ever tested positive for BRCA2 (either of two tumor supressor genes that in mutated form tend to be associated with an increased risk of certain cancers and especially	BRCA2 gene indicator	genes, BRCA2	Finkelstein Rare Cancers Registry Survey	PRISM	question	CAP to provide	en	

								breast and ovarian canc								
Have any of your family members ever tested positive for HNPCC (Hereditary Nonpolyposis Colon Cancer, also called Lynch Syndrome) - linked to colon, uterine, endometrial and other cancers?	☐ Yes<br>☐ Unknown<br>☐ No		ques tion	single select	Genetic testing	pati ent	01/06 /2011	Yes/no indicator to ask whether patient's family members ever tested positive to HNPCC (A group of autosomal-dominant inherited diseases in which colon cancer arises in discrete adenomas)	HNPCC gene indicator	Colorecta l Neoplas ms, Hereditar y Nonpolyp osis	Finkelste in Rare Cancers Registry Survey	PRI SM	ques tion	CAP to prov ide	e n	
Have you experienced episodes of infection requiring an ER visit or hospitalizato n and prolonged use of antibiotics?	☐ Yes<br>☐ Unknown<br>☐ No		ques tion	single-select	Medical history	pati ent	02/03 /2011	Yes/no indicator to ask if patient experienced infections requiring hospitalizatio n and prolonged use of antibiotics (Substances that reduce the growth or reproduction of bacteria)	infection indicator	anti-bacterial agents	Bone Marrow Failure Consorti um	PRI SM	ques tion	CAP to prov ide	e n	
If you have	☐ Site of		ques	single-	Medical	pati	02/03	Text	date and site	anti-	Bone	PRI	ques	CAP	e	

A Sample of PRISM Questions and Selected Metadata

experienced episodes of infection requiring an ER visit or hospitalization and prolonged use of antibiotics, please specify date and site	infection<br>□ Date of infection		tion	select	history	ent	/2011	indicator to specify date and site of infections requiring hospitalization and prolonged use of antibiotics	indicator	bacterial agents	Marrow Failure Consortium	SM	tion	to provide	n	
Have any of your family members ever tested positive for a genetic mutation related to cancer risk?	□ Yes<br>□ Unknown<br>□ No		question	single select	Genetic testing	patient	01/06/2011	Yes/no indicator to ask whether patient or family members ever tested positive for a genetic mutation related to cancer risk (testing for certain alleles, mutations, genotypes, or karyotypes that are associated with genetic traits, heritable diseases, or	family genetic mutation indicator	genetic testng	Finkelstein Rare Cancers Registry Survey	PRISM	question	CAP to provide	en	

Have any of your family members ever tested positive for FAP (Familial Adenomatous Polyposis, involves APC or MYH genes) - linked to multiple colon polyps, colon cancer?	☐ Yes<br>☐ Unknown<br>☐ No		question	single select	Genetic testing	patient	01/06/2011	Yes/no indicator to ask whether patient's family members ever tested positive for FAP (a disease of the large intestine that is marked by the formation of numerous adenomatous polyps which typically become malignant if left untreated, that may be either a	FAP gene indicator	Adenomatous Polyposis Coli	Finkelstein Rare Cancers Registry Survey	PRISM	question	CAP to provide	en	
Have any of your family members ever tested positive for PTEN - linked to Cowden Syndrome, breast cancer,	☐ Yes<br>☐ Unknown<br>☐ No		question	single select	Genetic testing	patient	01/06/2011	Yes/no indicator to ask whether patient's family members ever tested positive for PTEN (a benign, focal malformatio	PTEN gene indicator	Hamartoma Syndrome, Multiple	Finkelstein Rare Cancers Registry Survey	PRISM	question	CAP to provide	en	

thyroid tumors, and other cancers?								n that resembles a neoplasm in the tissue of its origin)								
Have any of your family members ever tested positive for Von Hippel-Lindau - a rare, genetic multi-system disorder characterized by the abnormal growth of tumors in certain parts of the body?	☐ Yes<br>☐ Unknown<br>☐ No		question	single select	Genetic testing	patient	01/06/2011	Yes/no indicator to ask whether patient's family members ever tested positive for Von Hippel-Lindau (a rare, genetic multi-system disorder characterized by the abnormal growth of tumors in certain parts of the body)	Von Hippel-Landau disorder indicator	Von Hippel-Lindau Disease	Finkelstein Rare Cancers Registry Survey	PRISM	question	CAP to provide	en	
If a family member tested positive for other genes not listed above, please indicate them here.	☐ Text field		question	single select	Genetic testing	patient	01/06/2011	Text indicator to signify other genetic tests patient's family tested positive for	Other gene indicator	genetic testing	Finkelstein Rare Cancers Registry Survey	PRISM	question	CAP to provide	en	
Did your father's mother ever			question	single select	Family history	patient	01/06/2011	Yes/no indicator to ask whether	Paternal grandmother second	breast neoplasms;	Rare Cancer Genetics	PRISM	question	CAP to prov	en	

receive a second diagnosis of breast cancer?								patient's paternal grandmother r ever received a second diagnosis of breast cancer	diagnosis of breast cancer indicator	diagnosis ;	Registry			ide		
If your father's mother ever received a second diagnosis of breast cancer, was it in the same breast as the first or the opposite?			ques tion	single select	Family history	pati ent	01/06 /2011	Text indicator to state whether patient's paternal grandfmothe r's second diagnosis of breast cancer was in the same or opposite breast	Second diagnosis of paternal grandmother 's breast cancer same or opposite breast indicator	breast neoplasm s; diagnosis ;	Rare Cancer Genetics Registry	PRI SM	ques tion	CAP to prov ide	e n	
What is the site of your biopsy?	☐ Text field		ques tion	single select	Diagnosi s details	pati ent	01/06 /2011	Text indicator to state site of biopsy	Site of biopsy indicator	Histiocyt osis, Langerha ns cell; diagnosis ; biopsy	Histiocyt e Society	PRI SM	ques tion	CAP to prov ide	e n	
What are your low risk organs?	☐ Bone (unifocal /multifoc al/special site)<br>☐ Skin<br>☐ Gastro-intestinal<br>☐ Other		ques tion	multi select	Diagnosi s details	pati ent	01/06 /2011	Text indicator to state low risk organs	Low risk organs indicator	Histiocyt osis, Langerha ns cell; diagnosis ; biopsy	Histiocyt e Society	PRI SM	ques tion	CAP to prov ide	e n	

	(specify)<br>☐ Lymph nodes<br>☐ Hypothalamic-pituitary region<br>☐ Nervous system<br>☐ Genital mucosa															
Primary Cancer Type	☐ Text field		question	single select	Disease specific medical history	patient	01/06/2011	Text indicator to signify primary cancer type	Primary cancer type indicator	neoplasms	Rare Cancer Genetics Registry	PRISM	question	CAP to provide	en	
If your father ever received a second diagnosis of breast cancer, was it in the same breast as the first or the opposite?	☐ Opposite breast<br>☐ Same breast		question	single select	Family history	patient	01/06/2011	Text indicator to signify whether patient's father's second breast cancer diagnosis was in the same or opposite breast	Father's second diagnosis same or opposite breast indicator	breast neoplasms; diagnosis;	Rare Cancer Genetics Registry	PRISM	question	CAP to provide	en	
Did your mother ever receive a second diagnosis of breast cancer?	☐ Yes<br>☐ Unknown<br>☐ No		question	single select	Family history	patient	01/06/2011	Yes/no indicator to ask whether patient's mother ever received a second diagnosis of	Mother second diagnosis of breast cancer indicator	breast neoplasms; diagnosis;	Rare Cancer Genetics Registry	PRISM	question	CAP to provide	en	

A Sample of PRISM Questions and Selected Metadata

								breast cancer								
If your mother received a second diagnosis of breast cancer, was it in the same breast as the first or the opposite?	☐ Opposite breast<br>☐ Same breast		question	single select	Family history	patient	01/06/2011	Text indicator to state whether patient's mother's second diagnosis of breast cancer was in the same or opposite breast	Mother's second diagnosis with breast cancer same breast or opposite indicator	breast neoplasms; diagnosis;	Rare Cancer Genetics Registry	PRISM	question	CAP to provide	en	
Did you have a LDL (Low-density lipoprotein cholesterol) test?	☐ Yes<br>☐ Not done<br>☐ Unknown<br>☐ No		question	single-select	Laboratory test results	patient	03/01/2011	Yes/no indicator to ask if patient had LDL (Low-density lipoprotein) test (LDL carries cholesterol in the blood. It is called "bad cholesterol" and is an indicator of hardening of the arteries)	LDL (Low-density lipoprotein) test indicator	liver function tests	Rare Diseases Clinical Research Network	PRISM	question		en	
What was the result of the LDL (Low-density lipoprotein cholesterol)	☐ Number field	mmol/L	question	number	Laboratory test results	patient	03/01/2011	Result value of LDL (Low-density lipoprotein) test	LDL (Low-density lipoprotein) test value	liver function tests	Rare Diseases Clinical Research Network	PRISM	question		en	

test?																
Was the result of the LDL (Low-density lipoprotein cholesterol) test within normal range?	☐ Yes<br>☐ Unknown<br>☐ No		question	single-select	Laboratory test results	patient	03/01/2011	Yes/no indicator to ask if patient's LDL (low-density lipoprotein) test results are within normal range (Less than 100 mg/dL (2.59 mmol/L) â€” Optimal. Higher levels can indicate risks for heart disease or other disorders)	LDL (Low-density lipoprotein) test, normal range indicator	liver function tests	Rare Diseases Clinical Research Network	PRISM	question		en	
Did you have a HDL (High-density lipoprotein cholesterol) test?	☐ Yes<br>☐ Not done<br>☐ Unknown<br>☐ No		question	single-select	Laboratory test results	patient	03/01/2011	Yes/no I ndicator to ask if patient had HDL (high-density lipoprotein) test (HDL-C consists primarily of protein with a small amount of cholesterol. It is considered	HDL (High-density lipoprotein) test value	liver function tests	Rare Diseases Clinical Research Network	PRISM	question		en	

								to be beneficial because it removes excess cholesterol from tissues and carries it to the								
What was the result of the HDL (High-density lipoprotein cholesterol) test?	☐ Number field	mmol/L	question	number	Laboratory test results	patient	03/01/2011	Value result for HDL (high-density lipoprotein)	HDL (High-density lipoprotein) test value	liver function tests	Rare Diseases Clinical Research Network	PRISM	question		en	
Was the result of the HDL (High-density lipoprotein cholesterol) test within normal range?	☐ Yes<br>☐ Unknown<br>☐ No		question	single-select	Laboratory test results	patient	03/01/2011	Yes/no indicator to ask if patient's HDL (high-density lipoprotein) results were within normal range (A typical level of HDL-C is between 40-50 mg/dL (1.0-1.3 mmol/L) for men and between 50-59 mg/dl (1.3-1.5	HDL (High-density lipoprotein) test, normal range indicator	liver function tests	Rare Diseases Clinical Research Network	PRISM	question		en	

								mmol/L) for women and is associated with averag								
What was the result of the D-dimer test?	☐ Negative<br>☐ Positive		question	number	Laboratory test results	patient	03/01/2011	Value result of D-dimer test	D-dimer test value	blood coagulation tests	Rare Diseases Clinical Research Network	PRISM	question		en	
What was the result of the urine pregnancy test?	☐ Negative<br>☐ Positive		question	single-select	Laboratory test results	patient	03/01/2011	status of urine pregnancy test (positive or negative)	urine pregnancy test status	pregnancy tests	Rare Diseases Clinical Research Network	PRISM	question		en	
During the last week, have you experienced eyes that are sensitive to light?	☐ Yes<br>☐ Unknown<br>☐ No		question	single-select	disease specific symptoms	patient	02/21/2011	Yes/no indiator to ask if patient's eyes are sensitive to light (photophobia is an abnormal sensitivity to light. This may occur as a manifestation of eye diseases, migraine, and other disorders)	light sensitivity in eyes	photophobia	Immune Mediated Disorders After Allogenic HCT	PRISM	question	CAP to assign	en	
Last week, I	☐ all of the		scal	single-	activity	chil	02/28	Enumerated	child's ability	activities			scal	CAP	e	

sat on the floor (eg at a school assembly or watching TV)	time<br>□ most of the time<br>□ I did not have any reason to sit on the floor<br>□ once in a while<br>□ none of the time<br>□ sometim es		e	select		d pati ent	/2011	list to indicate frequency child was able to sit on the floor	to sit on floor	of daily living			e	to assi gn	n	
What is your BMI (Body Mass Index?)	□ Number		ques tion	single-select	Anthrop ometry	pati ent	02/16 /2011	Number indicator to signify patient's body mass index (An indicator of body density as determined by the relationship of body weight to body height. The normal range is 18.5 to 24.9. A score of 30 or higher is obese.)	BMI indicator	body mass index	Brain Vascular Malform ation Consorti um	PRI SM	ques tion	CAP to assi gn	e n	
If your mother's father ever	□ Opposite breast<br>□ Same		ques tion	single select	Family history	pati ent	01/06 /2011	Text indicator to state	Site of second diagnosis of	breast neoplasm s;	Rare Cancer Genetics	PRI SM	ques tion	CAP to prov	e n	

received a second diagnosis of breast cancer, was it in the same breast as the first or the opposite?	breast							whether patient's maternal grandfather's second diagnosis of breast cancer was in the same or opposite breast	maternal grandfather's breast cancer indicator	diagnosis;	Registry			ide		
In the past year, has your child started or ended sensory integration therapy?	☐ Yes<br>☐ Continues to receive<br>☐ Unknown<br>☐ No		question	single-select	Child behavior	patient	01/12/2011	Yes/no indiator to ask if child started or ended sensory integration therapy in the past year (problems with their sense of touch, smell, hearing, taste and/or sight. Along with this will often be difficulties in movement, coordination and sensing where	sensory integration therapy indicator	sensory integration therapy	Angelman, Rett, Prader-Willi Consortium	PRISM	question	CAP to provide	en	
If your child started sensory integration	☐ Date field		question		Child behavior	patient	01/12/2011	Date indicator to signify when child started	sensory integration therapy indicator	sensory integration therapy	Angelman, Rett, Prader-Willi	PRISM	question	CAP to provide	en	

therapy, give the date								sensory integration therapy (problems with their sense of touch, smell, hearing, taste and/or sight. Along with this will often be difficulties in movement, coordination and sensing where one's body is in a gi			Consortium					
If your child ended sensory integration therapy, give the date	□ Date field		question		Child behavior	patient	01/12/2011	Date indicator to signify when child ended sensory integration therapy (problems with their sense of touch, smell, hearing, taste and/or sight. Along with this will often be difficulties in movement, coordination	sensory integration therapy indicator	sensory integration therapy	Angelman, Rett, Prader-Willi Consortium	PRISM	question	CAP to provide	en	

A Sample of PRISM Questions and Selected Metadata

								and sensing where one's body is in a give								
How long did sensory integration therapy last?	Number field	Months /years	ques tion		Child behavior	pati ent	01/12 /2011	Number indicator in months/year s to signify how long sensory integration therapy lasted (problems with their sense of touch, smell, hearing, taste and/or sight. Along with this will often be difficulties in movement, coordination and sensing where one's	sensory integration therapy indicator	sensory integratio n therapy	Angelma n, Rett, Prader-Willi Consorti um	PRI SM	ques tion	CAP to prov ide	e n	
At what age did your child become overweight (as determined by your physician)?	Numeric Value	Years or month s	ques tion	single-select	child history	pati ent	01/04 /2011	Age at which a child was diagnosed as overweight (body weight that is above certain standard of acceptable or desirable weight. In	overweight age	overweig ht	Angelma n, Rett, Prader-Willi Syndro mes Consorti um	PRI SM	ques tion	CAP to prov ide	e n	

								the scale of body mass index, overweight is defined as having a BMI of 25.0-29.9 kg/m2), as reported by the parent								
Your child's length at the time s/he was considered to be overweight	☐ Numeric Value	centimeters	question	single-select	child history; anthropometry	patient	01/04/2011	Body length (in centimeters) at the time of diagnosis of overweight (body weight that is above certain standard of acceptable or desirable weight. In the scale of body mass index, overweight is defined as having a BMI of 25.0-29.9 kg/m2), as reported by t	overweight measurement, length	body height	Angelman, Rett, Prader-Willi Syndromes Consortium	PRISM	question	CAP to provide	en	
During what time of the year is your sun	☐ Spring<br>☐ Summer<br>☐ Unknown		question	Single-select	Disorder-Specific Information:	patient	02/15/2011	Enumerated list to indicate at what time of	time of year for worst sun sensitivity indicator	photophobia; signs and symptom	Porphyria Rare Disease Clinical	PRISM	question	CAP to provide	en	

sensitivity the worst?	☐ Winter<br>☐ Fall				Congenital Erythropoietic Porphyria (CEP)			the year is the patient's sun sensitivity is the worst		s; porphyria, erythropoietic	Research Consortium					
How many episodes of sun sensitivity do you typically experience in a year?	☐ 1-5<br>☐ 5-10<br>☐ Unknown<br>☐ More than 15<br>☐ 10-15		question	Single-select	Disorder-Specific Information: Congenital Erythropoietic Porphyria (CEP)	patient	02/15/2011	Enumerated list to indicate the number of episodes of sun sensitivity the patient typically experiences in a year	number of yearly episodes of sun sensitivity indicator	photophobia; signs and symptoms; porphyria, erythropoietic	Porphyria Rare Disease Clinical Research Consortium	PRISM	question	CAP to provide	en		
Have you avoided spring outdoor sports during the daytime due to sun sensitivity?	☐ Yes<br>☐ Unknown<br>☐ No		question	Single-select	Disorder-Specific Information: Congenital Erythropoietic Porphyria (CEP)	patient	02/15/2011	Yes/no indicator to ask if the patient has avoided spring outdoor sports during the daytime due to sun sensitivity	avoidance of spring outdoor sports during the daytime indicator	photophobia; health behavior	Porphyria Rare Disease Clinical Research Consortium	PRISM	question	CAP to provide	en		
Have you avoided summer outdoor sports during the daytime due to sun sensitivity?	☐ Yes<br>☐ Unknown<br>☐ No		question	Single-select	Disorder-Specific Information: Congenital Erythropoietic Porphyria (CEP)	patient	02/15/2011	Yes/no indicator to ask if the patient has avoided summer outdoor sports during the daytime due to sun	avoidance of summer outdoor sports during the daytime indicator	photophobia; health behavior	Porphyria Rare Disease Clinical Research Consortium	PRISM	question	CAP to provide	en		

								sensitivity								
Have you avoided fall outdoor sports during the daytime due to sun sensitivity?	☐ Yes<br>☐ Unknown<br>☐ No		question	Single-select	Disorder-Specific Information: Congenital Erythropoietic Porphyria (CEP)	patient	02/15/2011	Yes/no indicator to ask if the patient has avoided fall outdoor sports during the daytime due to sun sensitivity	avoidance of fall outdoor sports during the daytime indicator	photophobia; health behavior	Porphyria Rare Disease Clinical Research Consortium	PRISM	question	CAP to provide	en	
Have you avoided winter outdoor sports during the daytime due to sun sensitivity?	☐ Yes<br>☐ Unknown<br>☐ No		question	Single-select	Disorder-Specific Information: Congenital Erythropoietic Porphyria (CEP)	patient	02/15/2011	Yes/no indicator to ask if the patient has avoided winter outdoor sports during the daytime due to sun sensitivity	avoidance of winter outdoor sports during the daytime indicator	photophobia; health behavior	Porphyria Rare Disease Clinical Research Consortium	PRISM	question	CAP to provide	en	
Have you avoided outdoor gardening during the daytime due to sun sensitivity?	☐ Yes<br>☐ Unknown<br>☐ No		question	Single-select	Disorder-Specific Information: Congenital Erythropoietic Porphyria (CEP)	patient	02/15/2011	Yes/no indicator to ask if the patient has avoided outdoor gardening during the daytime due to sun sensitivity	avoidance of outdoor gardening during the daytime indicator	photophobia; health behavior	Porphyria Rare Disease Clinical Research Consortium	PRISM	question	CAP to provide	en	
Have you avoided outdoor sunbathing	☐ Yes<br>☐ Unknown<br>☐ No		question	Single-select	Disorder-Specific Information:	patient	02/15/2011	Yes/no indicator to ask if the patient has	avoidance of outdoor sunbathing during the	photophobia; health behavior	Porphyria Rare Disease Clinical	PRISM	question	CAP to provide	en	

during the daytime due to sun sensitivity?					Congenital Erythropoietic Porphyria (CEP)			avoided outdoor sunbathing during the daytime due to sun sensitivity	daytime indicator		Research Consortium					
Have you ever had jaundice (yellow appearance of eyes and skin) after an episode of sun sensitivity?	☐ Yes<br>☐ Unknown<br>☐ No		question	Single-select	Disorder-Specific Information: Erythropoietic Porphyria (EP)	patient	02/15/2011	Yes/no indicator to ask if the patient had jaundice after sun exposure	jaundice as a result of sun exposure indicator	jaundice; photophobia	Porphyria Rare Disease Clinical Research Consortium	PRISM	question	CAP to provide	en	
How many times have you had jaundice (yellow appearance of eyes and skin) after an episode of sun sensitivity?	☐ Unknown<br>☐ Number field	number	question	Number	Disorder-Specific Information: Erythropoietic Porphyria (EP)	patient	02/15/2011	number of times patient had jaundice after sun exposure	frequency of jaundice as a result of sun exposure indicator	jaundice; photophobia	Porphyria Rare Disease Clinical Research Consortium	PRISM	question	CAP to provide	en	
How long does it typically take to recover from an episode of sun sensitivity?	☐ Less than 3 hours<br>☐ 3-24 hours<br>☐ More than 7 days<br>☐ 4-7 days<br>☐ 1-3 days		question	Single-select	Disorder-Specific Information: Erythropoietic Porphyria (EP)	patient	02/15/2011	Enumerated list to indicate length of time to recover after sun exposure	length of time to recover from an episode of sun sensitivity indicator	photophobia	Porphyria Rare Disease Clinical Research Consortium	PRISM	question	CAP to provide	en	

A Sample of PRISM Questions and Selected Metadata

Do you use opaque sunscreen for protection from sun sensitivity?	☐ Yes<br>☐ Unknown<br>☐ No		question	Single-select	Disorder-Specific Information: Erythropoietic Porphyria (EP)	patient	02/15/2011	Yes/no indicator to ask if the patient uses opaque sunscreen for sun protection	opaque sunscreen indicator	sunscreening agents	Porphyria Rare Disease Clinical Research Consortium	PRISM	question	CAP to provide	en	
Do you use bronzers or theatrical makeup for protection from sun sensitivity?	☐ Yes<br>☐ Unknown<br>☐ No		question	Single-select	Disorder-Specific Information: Erythropoietic Porphyria (EP)	patient	02/15/2011	Yes/no indicator to ask if the patient uses bronzers or theatrical makeup for sun protection	bronzers or theatrical make up indicator	sunscreening agents	Porphyria Rare Disease Clinical Research Consortium	PRISM	question	CAP to provide	en	
Did you not select another occupation because of your sun sensitivity?	☐ Yes<br>☐ Unknown<br>☐ No		question	Single-select	Disorder-Specific Information: Congenital Erythropoietic Porphyria (CEP)	patient	02/15/2011	Yes/no indicator to ask if the patient did not select another occupation because of sun sensitivity	occupation not selected due to sun sensitivity indicator	photophobia; health behavior	Porphyria Rare Disease Clinical Research Consortium	PRISM	question	CAP to provide	en	
Did you ever quit a job because of your sun sensitivity?	☐ Yes<br>☐ Unknown<br>☐ No		question	Single-select	Disorder-Specific Information: Congenital Erythropoietic Porphyria (CEP)	patient	02/15/2011	Yes/no indicator to ask if the patient ever quit a job because of sun sensitivity	quit job due to sun sensitivity indicator	photophobia; health behavior	Porphyria Rare Disease Clinical Research Consortium	PRISM	question	CAP to provide	en	

Describe the effect of the sun sensitivity on your lifestyle?	☐ No inconvenience<br>☐ Unknown<br>☐ Major inconvenience, preventing you from doing many things that you wanted to do<br>☐ Mild inconvenience		question	Single-select	Disorder-Specific Information: Congenital Erythropoietic Porphyria (CEP)	patient	02/15/2011	Enumerated list to indicate the effect of sun sensitivity on the patient's lifestyle	sun sensitivity effect on lifestyle indicator	photophobia; health behavior	Porphyria Rare Disease Clinical Research Consortium	PRISM	question	CAP to provide	en	
Have you ever been depressed (depressive states usually of moderate intensity in contrast with major depression present in neurotic and psychotic disorders)?	☐ Yes<br>☐ Unknown<br>☐ No		question	Single-select	Disorder-Specific Information: Congenital Erythropoietic Porphyria (CEP)	patient	02/15/2011	Yes/no indicator to ask if the patient has ever been depressed. Depression constitutes depressive states usually of moderate intensity in contrast with major depression present in neurotic and psychotic	depression indicator	Depression	Porphyria Rare Disease Clinical Research Consortium	PRISM	question	CAP to provide	en	

								disorders.								
Do you think that your sun sensitivity contributed to your depression?	☐ Yes<br>☐ Unknown<br>☐ No		question	Single-select	Disorder-Specific Information: Congenital Erythropoietic Porphyria (CEP)	patient	02/15/2011	Yes/no indicator to ask if the patient thinks that sun sensitivity contributed to his/her depression	sun sensitivity contributing to depression indicator	photophobia; depression	Porphyria Rare Disease Clinical Research Consortium	PRISM	question	CAP to provide	en	
Are blisters on your face a chronic skin change you have experienced because of sun sensitivity?	☐ Yes<br>☐ Unknown<br>☐ No		question	Single-select	Disorder-Specific Information: Erythropoietic Porphyria (EP)	patient	02/15/2011	Yes/no indicator to ask if the patient has blisters on face due to sun sensitivity	blisters on face indicator	blisters; photophobia	Porphyria Rare Disease Clinical Research Consortium	PRISM	question	CAP to provide	en	
Are blisters on your ears a chronic skin change you have experienced because of sun sensitivity?	☐ Yes<br>☐ Unknown<br>☐ No		question	Single-select	Disorder-Specific Information: Erythropoietic Porphyria (EP)	patient	02/15/2011	Yes/no indicator to ask if the patient has blisters on ears due to sun sensitivity	blisters on ears indicator	blisters; photophobia	Porphyria Rare Disease Clinical Research Consortium	PRISM	question	CAP to provide	en	
Are blisters on your legs a chronic skin change you have experienced because of sun	☐ Yes<br>☐ Unknown<br>☐ No		question	Single-select	Disorder-Specific Information: Erythropoietic Porphyria (EP)	patient	02/15/2011	Yes/no indicator to ask if the patient has blisters on legs due to sun sensitivity	blisters on legs indicator	blisters; photophobia	Porphyria Rare Disease Clinical Research Consortium	PRISM	question	CAP to provide	en	

sensitivity?																
Are blisters on your arms a chronic skin change you have experienced because of sun sensitivity?	☐ Yes<br>☐ Unknown<br>☐ No		question	Single-select	Disorder-Specific Information: Erythropoietic Porphyria (EP)	patient	02/15/2011	Yes/no indicator to ask if the patient has blisters on arms due to sun sensitivity	blisters on arms indicator	blisters; photophobia	Porphyria Rare Disease Clinical Research Consortium	PRISM	question	CAP to provide	en	
Is redness on your hands a chronic skin change you have experienced because of sun sensitivity?	☐ Yes<br>☐ Unknown<br>☐ No		question	Single-select	Disorder-Specific Information: Erythropoietic Porphyria (EP)	patient	02/15/2011	Yes/no indicator to ask if the patient has redness on hands due to sun sensitivity	redness on hands indicator	skin manifestations; photophobia	Porphyria Rare Disease Clinical Research Consortium	PRISM	question	CAP to provide	en	
Is redness on your feet a chronic skin change you have experienced because of sun sensitivity?	☐ Yes<br>☐ Unknown<br>☐ No		question	Single-select	Disorder-Specific Information: Erythropoietic Porphyria (EP)	patient	02/15/2011	Yes/no indicator to ask if the patient has redness on feet due to sun sensitivity	redness on feet indicator	skin manifestations; photophobia	Porphyria Rare Disease Clinical Research Consortium	PRISM	question	CAP to provide	en	
Is redness on your face a chronic skin change you have experienced because of	☐ Yes<br>☐ Unknown<br>☐ No		question	Single-select	Disorder-Specific Information: Erythropoietic Porphyri	patient	02/15/2011	Yes/no indicator to ask if the patient has redness on face due to sun	redness on face indicator	skin manifestations; photophobia	Porphyria Rare Disease Clinical Research Consorti	PRISM	question	CAP to provide	en	

sun sensitivity?					a (EP)			sensitivity			um					
Is redness on your ears a chronic skin change you have experienced because of sun sensitivity?	☐ Yes<br>☐ Unknown<br>☐ No		question	Single-select	Disorder-Specific Information: Erythropoietic Porphyria (EP)	patient	02/15/2011	Yes/no indicator to ask if the patient has redness on ears due to sun sensitivity	redness on ears indicator	skin manifestations; photophobia	Porphyria Rare Disease Clinical Research Consortium	PRISM	question	CAP to provide	en	
Is redness on your legs a chronic skin change you have experienced because of sun sensitivity?	☐ Yes<br>☐ Unknown<br>☐ No		question	Single-select	Disorder-Specific Information: Erythropoietic Porphyria (EP)	patient	02/15/2011	Yes/no indicator to ask if the patient has redness on legs due to sun sensitivity	redness on legs indicator	skin manifestations; photophobia	Porphyria Rare Disease Clinical Research Consortium	PRISM	question	CAP to provide	en	
Is redness on your arms a chronic skin change you have experienced because of sun sensitivity?	☐ Yes<br>☐ Unknown<br>☐ No		question	Single-select	Disorder-Specific Information: Erythropoietic Porphyria (EP)	patient	02/15/2011	Yes/no indicator to ask if the patient has redness on arms due to sun sensitivity	redness on arms indicator	skin manifestations; photophobia	Porphyria Rare Disease Clinical Research Consortium	PRISM	question	CAP to provide	en	
Is swelling in your hands a chronic skin change you have experienced because of	☐ Yes<br>☐ Unknown<br>☐ No		question	Single-select	Disorder-Specific Information: Erythropoietic Porphyri	patient	02/15/2011	Yes/no indicator to ask if the patient has swelling in hands due to sun	swelling in hands indicator	inflammation; photophobia	Porphyria Rare Disease Clinical Research Consorti	PRISM	question	CAP to provide	en	

sun sensitivity?					a (EP)			sensitivity			um					
Is swelling in your feet a chronic skin change you have experienced because of sun sensitivity?	☐ Yes<br>☐ Unknown<br>☐ No		question	Single-select	Disorder-Specific Information: Erythropoietic Porphyria (EP)	patient	02/15/2011	Yes/no indicator to ask if the patient has swelling in feet due to sun sensitivity	swelling in feet indicator	inflammation; photophobia	Porphyria Rare Disease Clinical Research Consortium	PRISM	question	CAP to provide	en	
When did sun sensitivity first occur?	☐ Infancy<br>☐ Childhood<br>☐ Unknown<br>☐ Adult<br>☐ Teenager		question	Single-select	Disorder-Specific Information: Erythropoietic Porphyria (EP)	patient	02/15/2011	Enumerated list to indicate first occurrence of sun sensitivity	first occurrence of sun sensitivity indicator	photophobia	Porphyria Rare Disease Clinical Research Consortium	PRISM	question	CAP to provide	en	
How old were you when sun sensitivity first occurred?	☐ Unknown<br>☐ Number field	age	question	Number	Disorder-Specific Information: Erythropoietic Porphyria (EP)	patient	02/15/2011	Age (in years) that sun sentivity first occurred	age at first occurrence of sun sensitivity indicator	photophobia	Porphyria Rare Disease Clinical Research Consortium	PRISM	question	CAP to provide	en	
Would you describe the discomfort on sun-exposed skin as tingling?	☐ Yes<br>☐ Unknown<br>☐ No		question	Single-select	Disorder-Specific Information: Erythropoietic Porphyria (EP)	patient	02/15/2011	Yes/no indicator to ask if the patient has tingling when exposed to sun	tingling indicator	paresthesia; photophobia	Porphyria Rare Disease Clinical Research Consortium	PRISM	question	CAP to provide	en	
Would you describe the	☐ Yes<br>☐ Unknown		question	Single-select	Disorder-Specific	patient	02/15/2011	Yes/no indicator to	itching indicator	pruritis; photopho	Porphyria Rare	PRISM	question	CAP to	en	

discomfort on sun-exposed skin as itching?	☐ No				Information: Erythropoietic Porphyria (EP)			ask if the patient has itching when exposed to sun		bia	Disease Clinical Research Consortium			provide	
Would you describe the discomfort on sun-exposed skin as stinging?	☐ Yes<br>☐ Unknown<br>☐ No		question	Single-select	Disorder-Specific Information: Erythropoietic Porphyria (EP)	patient	02/15/2011	Yes/no indicator to ask if the patient has stinging when exposed to sun	stinging indicator	pain; photophobia	Porphyria Rare Disease Clinical Research Consortium	PRISM	question	CAP to provide	en
Would you describe the discomfort on sun-exposed skin as burning?	☐ Yes<br>☐ Unknown<br>☐ No		question	Single-select	Disorder-Specific Information: Erythropoietic Porphyria (EP)	patient	02/15/2011	Yes/no indicator to ask if the patient burning when exposed to sun	burning indicator	pain; photophobia	Porphyria Rare Disease Clinical Research Consortium	PRISM	question	CAP to provide	en
What other term would you use to describe the discomfort on sun-exposed skin?	☐ Unknown<br>☐ Text field	text	question	Text	Disorder-Specific Information: Erythropoietic Porphyria (EP)	patient	02/15/2011	Text specifying other effects of sun exposure	other discomfort indicator	photophobia	Porphyria Rare Disease Clinical Research Consortium	PRISM	question	CAP to provide	en
Do you use film placed on your car windows for protection from sun sensitivity?	☐ Yes<br>☐ Unknown<br>☐ No		question	Single-select	Disorder-Specific Information: Erythropoietic Porphyri	patient	02/15/2011	Yes/no indicator to ask if the patient uses film placed on car windows for	film placed on car windows indicator	sunscreening agents	Porphyria Rare Disease Clinical Research Consorti	PRISM	question	CAP to provide	en

					a (EP)			sun protection			um					
What else do you use for protection from sun sensitivity?	☐ Unknown<br>☐ Text field	text	question	Text	Disorder-Specific Information: Erythropoietic Porphyria (EP)	patient	02/15/2011	Text specifying other methods of sun protection used by patient	other sun protection indicator	sunscreening agents	Porphyria Rare Disease Clinical Research Consortium	PRISM	question	CAP to provide	en	
How often do you use measures to protect from sun sensitivity?	☐ Never<br>☐ Less than half the time<br>☐ Unknown<br>☐ Always<br>☐ More than half the time		question	Single-select	Disorder-Specific Information: Erythropoietic Porphyria (EP)	patient	02/15/2011	Enumerated list to indicate frequency that sun protection is used.	frequency of use of sun protection measures indicator	sunscreening agents	Porphyria Rare Disease Clinical Research Consortium	PRISM	question	CAP to provide	en	
Does wind make the sun sensitivity worse?	☐ Yes<br>☐ Unknown<br>☐ No		question	Single-select	Disorder-Specific Information: Erythropoietic Porphyria (EP)	patient	02/15/2011	Yes/no indicator to ask the patient if the wind makes sun sensitivity worse	wind as a sun sensitivity trigger indicator	photophobia	Porphyria Rare Disease Clinical Research Consortium	PRISM	question	CAP to provide	en	
Does eating just before going outside make the sun sensitivity worse?	☐ Yes<br>☐ Unknown<br>☐ No		question	Single-select	Disorder-Specific Information: Erythropoietic Porphyria (EP)	patient	02/15/2011	Yes/no indicator to ask the patient if eating before going outside makes sun sensitivity worse	eating as a sun sensitivity trigger	photophobia	Porphyria Rare Disease Clinical Research Consortium	PRISM	question	CAP to provide	en	

A Sample of PRISM Questions and Selected Metadata

Does drinking alcohol before going outside make the sun sensitivity worse?	☐ Yes<br>☐ Unknown<br>☐ No		question	Single-select	Disorder-Specific Information: Erythropoietic Porphyria (EP)	patient	02/15/2011	Yes/no indicator to ask the patient if drinking alcohol before going outside makes sun sensitivity worse	drinking alcholo as a sun sensitivity triger indicator	photophobia	Porphyria Rare Disease Clinical Research Consortium	PRISM	question	CAP to provide	en	
Does exercise make the sun sensitivity worse?	☐ Yes<br>☐ Unknown<br>☐ No		question	Single-select	Disorder-Specific Information: Erythropoietic Porphyria (EP)	patient	02/15/2011	Yes/no indicator to ask the patient if exercise makes sun sensitivity worse	exercise as a sun sensitivity trigger indicator	photophobia	Porphyria Rare Disease Clinical Research Consortium	PRISM	question	CAP to provide	en	
Does being on water make the sun sensitivity worse?	☐ Yes<br>☐ Unknown<br>☐ No		question	Single-select	Disorder-Specific Information: Erythropoietic Porphyria (EP)	patient	02/15/2011	Yes/no indicator to ask the patient if being on water makes sun sensitivity worse	being on water as a sun sensivity trigger indicator	photophobia	Porphyria Rare Disease Clinical Research Consortium	PRISM	question	CAP to provide	en	
Does taking certain medications make the sun sensitivity worse?	☐ Yes<br>☐ Unknown<br>☐ No		question	Single-select	Disorder-Specific Information: Erythropoietic Porphyria (EP)	patient	02/15/2011	Yes/no indicator to ask the patient if taking certain medications makes sun sensitivity worse	certain medications as a sun sensitivity trigger indicator	photophobia	Porphyria Rare Disease Clinical Research Consortium	PRISM	question	CAP to provide	en	

A Sample of PRISM Questions and Selected Metadata

What medications make the sun sensitivity worse?	Unknown<br>Text field	text	question	Text	Disorder-Specific Information: Erythropoietic Porphyria (EP)	patient	02/15/2011	Text specifying medications that make sun sensitivity worse	particular medications that trigger sun sensitivity indicator	photophobia	Porphyria Rare Disease Clinical Research Consortium	PRISM	question	CAP to provide	en	
Do certain illnesses make the sun sensitivity worse?	Yes<br>Unknown<br>No		question	Single-select	Disorder-Specific Information: Erythropoietic Porphyria (EP)	patient	02/15/2011	Yes/no indicator to ask the patient if certain illnesses make sun sensitivity worse	certain illnesses as a sun sensitivity trigger indicator	photophobia	Porphyria Rare Disease Clinical Research Consortium	PRISM	question	CAP to provide	en	
What illnesses make the sun sensitivity worse?	Unknown<br>Text field	text	question	Text	Disorder-Specific Information: Erythropoietic Porphyria (EP)	patient	02/15/2011	Text specifying illnesses that make sun sensitivity worse	particular illnesses that trigger sun sensitivity indicator	photophobia	Porphyria Rare Disease Clinical Research Consortium	PRISM	question	CAP to provide	en	
What other factors make the sun sensitivity worse?	Unknown<br>Text field	text	question	Text	Disorder-Specific Information: Erythropoietic Porphyria (EP)	patient	02/15/2011	Text specifying other things that make sun sensitivity worse	other sun sensitivity trigger indicators	photophobia	Porphyria Rare Disease Clinical Research Consortium	PRISM	question	CAP to provide	en	
How did the sun sensitivity change	Better<br>Unknown<br>No change		question	Single-select	Disorder-Specific Information:	patient	02/15/2011	Enumerated list to indicate how sun	effect of pregnancy on sun sensitivity	photophobia; pregnancy	Porphyria Rare Disease Clinical	PRISM	question	CAP to provide	en	

during pregnancy?	☐ Worse				Erythropoietic Porphyria (EP)			sensitivity changed during pregnancy			Research Consortium					
Did taking beta-carotene capsules help prevent sun sensitivity?	☐ Yes<br>☐ Unknown<br>☐ No		question	Single-select	Disorder-Specific Information: Erythropoietic Porphyria (EP)	patient	02/15/2011	Yes/no indicator to ask the patient if beta-carotene capsules helped prevent sun sensitivity (beta Carotene is a carotenoid that is a precursor of Vitamin A)	beta-carotine to prevent sun sensitivity indicator	beta-Carotene; photophobia	Porphyria Rare Disease Clinical Research Consortium	PRISM	question	CAP to provide	en	
How many episodes of sun sensitivity typically occur in a year?	☐ 1-2<br>☐ 3-10<br>☐ More than 60<br>☐ 31-60<br>☐ 11-30		question	Single-select	Disorder-Specific Information: Erythropoietic Porphyria (EP)	patient	02/15/2011	Enumerated list to indicate number of yearly episodes of sun sensitivity	frequency of episodes of sun sensitivity indicator	photophobia	Porphyria Rare Disease Clinical Research Consortium	PRISM	question	CAP to provide	en	
What time of year is the worst for your sun sensitivity?	☐ Spring<br>☐ Winter<br>☐ Fall<br>☐ Summer		question	Single-select	Disorder-Specific Information: Erythropoietic Porphyria (EP)	patient	02/15/2011	Enumerated list of text codes and names to represent worst time of year for sun sensitivity	season for most severe sun sensitivity indicator	photophobia	Porphyria Rare Disease Clinical Research Consortium	PRISM	question	CAP to provide	en	
Do you use a	☐ Yes		ques	Single-	Disorder	pati	02/15	Yes/no	long sleeve	sunscree	Porphyri	PRI	ques	CAP	e	

long-sleeve shirt or top for protection from sun sensitivity?	☐ Unknown<br>☐ No		tion	select	-Specific Information: Erythropoietic Porphyria (EP)	ent	/2011	indicator to ask if the patient wears a long sleeve shirt or top for sun protection	shirt indicator	ning agents	a Rare Disease Clinical Research Consortium	SM	tion	to provide	n	
Do you use gloves for protection from sun sensitivity?	☐ Yes<br>☐ Unknown<br>☐ No		question	Single-select	Disorder-Specific Information: Erythropoietic Porphyria (EP)	patient	02/15/2011	Yes/no indicator to ask if the patient wears gloves for sun protection	gloves indicator	sunscreening agents	Porphyria Rare Disease Clinical Research Consortium	PRISM	question	CAP to provide	en	
Do you use a cap for protection from sun sensitivity?	☐ Yes<br>☐ Unknown<br>☐ No		question	Single-select	Disorder-Specific Information: Erythropoietic Porphyria (EP)	patient	02/15/2011	Yes/no indicator to ask if the patient wears a cap for sun protection	cap indicator	sunscreening agents	Porphyria Rare Disease Clinical Research Consortium	PRISM	question	CAP to provide	en	
Do you use sunglasses for protection from sun sensitivity?	☐ Yes<br>☐ Unknown<br>☐ No		question	Single-select	Disorder-Specific Information: Erythropoietic Porphyria (EP)	patient	02/15/2011	Yes/no indicator to ask if the patient wears sunglasses for sun protection	sunglasses indicator	sunscreening agents	Porphyria Rare Disease Clinical Research Consortium	PRISM	question	CAP to provide	en	
Do you use regular sunscreen for protection	☐ Yes<br>☐ Unknown<br>☐ No		question	Single-select	Disorder-Specific Information: Erythrop	patient	02/15/2011	Yes/no indicator to ask if the patient uses regular	regular sunscreen indicator	sunscreening agents	Porphyria Rare Disease Clinical Researc	PRISM	question	CAP to provide	en	

from sun sensitivity?					oietic Porphyria (EP)			sunscreen for sun protection			h Consortium					
Did taking cysteine help prevent sun sensitivity?	☐ Yes<br>☐ Unknown<br>☐ No		question	Single-select	Disorder-Specific Information: Erythropoietic Porphyria (EP)	patient	02/15/2011	Yes/no indicator to ask the patient if taking cysteine helped prevent sun sensitivity	cysteine to prevent sun sensitivity indicator	cysteine; photophobia	Porphyria Rare Disease Clinical Research Consortium	PRISM	question	CAP to provide	en	
Do you have chronic skin changes because of the sun sensitivity?	☐ Yes<br>☐ Unknown<br>☐ No		question	Single-select	Disorder-Specific Information: Erythropoietic Porphyria (EP)	patient	02/15/2011	Yes/no indicator to ask if the patient has chronic skin changes because of sun sensitivity	chronic skin changes indicator	skin manifestations; photophobia	Porphyria Rare Disease Clinical Research Consortium	PRISM	question	CAP to provide	en	
Are blisters on your hands a chronic skin change you have experienced because of sun sensitivity?	☐ Yes<br>☐ Unknown<br>☐ No		question	Single-select	Disorder-Specific Information: Erythropoietic Porphyria (EP)	patient	02/15/2011	Yes/no indicator to ask if the patient has blisters on hands due to sun sensitivity	blisters on hands indicator	blisters; photophobia	Porphyria Rare Disease Clinical Research Consortium	PRISM	question	CAP to provide	en	
Are blisters on your feet a chronic skin change you have experienced	☐ Yes<br>☐ Unknown<br>☐ No		question	Single-select	Disorder-Specific Information: Erythropoietic	patient	02/15/2011	Yes/no indicator to ask if patient has blisters on feet due to sun	blisters on feet indicator	blisters; photophobia	Porphyria Rare Disease Clinical Research	PRISM	question	CAP to provide	en	

because of sun sensitivity?					Porphyria (EP)			sensitivity			Consortium					
Is swelling in your face a chronic skin change you have experienced because of sun sensitivity?	☐ Yes<br>☐ Unknown<br>☐ No		question	Single-select	Disorder-Specific Information: Erythropoietic Porphyria (EP)	patient	02/15/2011	Yes/no indicator to ask if the patient has swelling in face due to sun sensitivity	swelling in face indicator	inflammation; photophobia	Porphyria Rare Disease Clinical Research Consortium	PRISM	question	CAP to provide	en	
Is swelling in your ears a chronic skin change you have experienced because of sun sensitivity?	☐ Yes<br>☐ Unknown<br>☐ No		question	Single-select	Disorder-Specific Information: Erythropoietic Porphyria (EP)	patient	02/15/2011	Yes/no indicator to ask if the patient has swelling in ears due to sun sensitivity	swelling in ears indicator	inflammation; photophobia	Porphyria Rare Disease Clinical Research Consortium	PRISM	question	CAP to provide	en	
Is swelling in your legs a chronic skin change you have experienced because of sun sensitivity?	☐ Yes<br>☐ Unknown<br>☐ No		question	Single-select	Disorder-Specific Information: Erythropoietic Porphyria (EP)	patient	02/15/2011	Yes/no indicator to ask if the patient has swelling in legs due to sun sensitivity	swelling in legs indicator	inflammation; photophobia	Porphyria Rare Disease Clinical Research Consortium	PRISM	question	CAP to provide	en	
Is swelling in your arms a chronic skin change you have experienced because of	☐ Yes<br>☐ Unknown<br>☐ No		question	Single-select	Disorder-Specific Information: Erythropoietic Porphyri	patient	02/15/2011	Yes/no indicator to ask if the patient has swelling in arms due to sun	swelling in arms indicator	inflammation; photophobia	Porphyria Rare Disease Clinical Research Consorti	PRISM	question	CAP to provide	en	

sun sensitivity?					a (EP)			sensitivity			um					
Are pigment changes on your legs a chronic skin change you have experienced because of sun sensitivity?	☐ Yes<br>☐ Unknown<br>☐ No		question	Single-select	Disorder-Specific Information: Erythropoietic Porphyria (EP)	patient	02/15/2011	Yes/no indicator to ask if the patient has pigment changes on legs due to sun sensitivity	pigment changes on legs indicator	photophobia; skin manifestations	Porphyria Rare Disease Clinical Research Consortium	PRISM	question	CAP to provide	en	
Are pigment changes on your arms a chronic skin change you have experienced because of sun sensitivity?	☐ Yes<br>☐ Unknown<br>☐ No		question	Single-select	Disorder-Specific Information: Erythropoietic Porphyria (EP)	patient	02/15/2011	Yes/no indicator to ask if the patient has pigment changes on arms due to sun sensitivity	pigment changes on arms indicator	photophobia; skin manifestations	Porphyria Rare Disease Clinical Research Consortium	PRISM	question	CAP to provide	en	
Did you select your current occupation because of your sun sensitivity?	☐ Yes<br>☐ Unknown<br>☐ Unemployed<br>☐ No		question	Single-select	Disorder-Specific Information: Erythropoietic Porphyria (EP)	patient	02/15/2011	Yes/no indicator to ask if the patient chose current occupation due to sun sensitivity	effect of sun sensitivity on occupation	photophobia; life style	Porphyria Rare Disease Clinical Research Consortium	PRISM	question	CAP to provide	en	
Did you ever quit a job because of your sun sensitivity?	☐ Yes<br>☐ Unknown<br>☐ No		question	Single-select	Disorder-Specific Information: Erythropoietic	patient	02/15/2011	Yes/no indicator to ask if the patient ever quit a job due to sun	effect of sun sensitivity on job	photophobia; life style	Porphyria Rare Disease Clinical Research	PRISM	question	CAP to provide	en	

					Porphyria (EP)			sensitivity			Consortium					
Describe the effect of the sun sensitivity on your lifestyle?	☐ No inconvenience<br>☐ Unknown<br>☐ Major inconvenience, preventing you from doing many things that you wanted to do<br>☐ Mild inconvenience		question	Single-select	Disorder-Specific Information: Erythropoietic Porphyria (EP)	patient	02/15/2011	Enumerated list to indicate effect of sun sensitivity on lifestyle	effect of sun sensitivity on lifestyle	photophobia; life style	Porphyria Rare Disease Clinical Research Consortium	PRISM	question	CAP to provide	en	
Do you think that your sun sensitivity contributed to your depression?	☐ Yes<br>☐ Unknown<br>☐ No		question	Single-select	Disorder-Specific Information: Erythropoietic Porphyria (EP)	patient	02/15/2011	Yes/no indicator to ask if the patient thinks sun sensitivity contributed to depression	the effect of sun sensitivity on depression	depression; photophobia	Porphyria Rare Disease Clinical Research Consortium	PRISM	question	CAP to provide	en	
Do you have sensitivity to fluorescent lights?	☐ Yes<br>☐ Unknown<br>☐ No		question	Single-select	Disorder-Specific Information: Erythropoietic Porphyria (EP)	patient	02/15/2011	Yes/no indicator to ask if the patient is sensitive to fluorescent lights	sensitivity to fluorescent lights indicator	photophobia	Porphyria Rare Disease Clinical Research Consortium	PRISM	question	CAP to provide	en	

A Sample of PRISM Questions and Selected Metadata

Is scarring on your hands a chronic skin change you have experienced because of sun sensitivity?	☐ Yes<br>☐ Unknown<br>☐ No		question	Single-select	Disorder-Specific Information: Erythropoietic Porphyria (EP)	patient	02/15/2011	Yes/no indicator to ask if the patient has scarring on hands due to sun sensitivity	scarring on hands indicator	cicatrix; photophobia	Porphyria Rare Disease Clinical Research Consortium	PRISM	question	CAP to provide	en	
Is scarring on your feet a chronic skin change you have experienced because of sun sensitivity?	☐ Yes<br>☐ Unknown<br>☐ No		question	Single-select	Disorder-Specific Information: Erythropoietic Porphyria (EP)	patient	02/15/2011	Yes/no indicator to ask if the patient has scarring on feet due to sun sensitivity	scarring on feet indicator	cicatrix; photophobia	Porphyria Rare Disease Clinical Research Consortium	PRISM	question	CAP to provide	en	
Is scarring on your face a chronic skin change you have experienced because of sun sensitivity?	☐ Yes<br>☐ Unknown<br>☐ No		question	Single-select	Disorder-Specific Information: Erythropoietic Porphyria (EP)	patient	02/15/2011	Yes/no indicator to ask if the patient has scarring on face due to sun sensitivity	scarring on face indicator	cicatrix; photophobia	Porphyria Rare Disease Clinical Research Consortium	PRISM	question	CAP to provide	en	
Is scarring on your ears a chronic skin change you have experienced because of sun sensitivity?	☐ Yes<br>☐ Unknown<br>☐ No		question	Single-select	Disorder-Specific Information: Erythropoietic Porphyria (EP)	patient	02/15/2011	Yes/no indicator to ask if the patient has scarring on ears due to sun sensitivity	scarring on ears indicator	cicatrix; photophobia	Porphyria Rare Disease Clinical Research Consortium	PRISM	question	CAP to provide	en	

Is scarring on your legs a chronic skin change you have experienced because of sun sensitivity?	☐ Yes<br>☐ Unknown<br>☐ No		question	Single-select	Disorder-Specific Information: Erythropoietic Porphyria (EP)	patient	02/15/2011	Yes/no indicator to ask if the patient has scarring on legs due to sun sensitivity	scarring on legs indicator	cicatrix; photophobia	Porphyria Rare Disease Clinical Research Consortium	PRISM	question	CAP to provide	en		
Is scarring on your arms a chronic skin change you have experienced because of sun sensitivity?	☐ Yes<br>☐ Unknown<br>☐ No		question	Single-select	Disorder-Specific Information: Erythropoietic Porphyria (EP)	patient	02/15/2011	Yes/no indicator to ask if the patient has scarring on arms due to sun sensitivity	scarring on arms indicator	cicatrix; photophobia	Porphyria Rare Disease Clinical Research Consortium	PRISM	question	CAP to provide	en		
Is crusty skin on your hands a chronic skin change you have experienced because of sun sensitivity?	☐ Yes<br>☐ Unknown<br>☐ No		question	Single-select	Disorder-Specific Information: Erythropoietic Porphyria (EP)	patient	02/15/2011	Yes/no indicator to ask if the patient has crusty skin on hands due to sun sensitivity	crusty skin on hands indicator	skin manifestations; photophobia	Porphyria Rare Disease Clinical Research Consortium	PRISM	question	CAP to provide	en		
Is crusty skin on your feet a chronic skin change you have experienced because of sun	☐ Yes<br>☐ Unknown<br>☐ No		question	Single-select	Disorder-Specific Information: Erythropoietic Porphyria (EP)	patient	02/15/2011	Yes/no indicator to ask if the patient has crusty skin on feet due to sun sensitivity	crusty skin on feet indicator	skin manifestations; photophobia	Porphyria Rare Disease Clinical Research Consortium	PRISM	question	CAP to provide	en		

sensitivity?																
Is crusty skin on your face a chronic skin change you have experienced because of sun sensitivity?	☐ Yes<br>☐ Unknown<br>☐ No		question	Single-select	Disorder-Specific Information: Erythropoietic Porphyria (EP)	patient	02/15/2011	Yes/no indicator to ask if the patient has crusty skin on face due to sun sensitivity	crusty skin on face indicator	skin manifestations; photophobia	Porphyria Rare Disease Clinical Research Consortium	PRISM	question	CAP to provide	en	
Is crusty skin on your ears a chronic skin change you have experienced because of sun sensitivity?	☐ Yes<br>☐ Unknown<br>☐ No		question	Single-select	Disorder-Specific Information: Erythropoietic Porphyria (EP)	patient	02/15/2011	Yes/no indicator to ask if the patient has crusty skin on ears due to sun sensitivity	crusty skin on ears indicator	skin manifestations; photophobia	Porphyria Rare Disease Clinical Research Consortium	PRISM	question	CAP to provide	en	
Is crusty skin on your legs a chronic skin change you have experienced because of sun sensitivity?	☐ Yes<br>☐ Unknown<br>☐ No		question	Single-select	Disorder-Specific Information: Erythropoietic Porphyria (EP)	patient	02/15/2011	Yes/no indicator to ask if the patient has crusty skin on legs due to sun sensitivity	crusty skin on legs indicator	skin manifestations; photophobia	Porphyria Rare Disease Clinical Research Consortium	PRISM	question	CAP to provide	en	
Is crusty skin on your arms a chronic skin change you have	☐ Yes<br>☐ Unknown<br>☐ No		question	Single-select	Disorder-Specific Information: Erythropoietic	patient	02/15/2011	Yes/no indicator to ask if the patient has crusty skin on arms due	crusty skin on arms indicator	skin manifestations; photophobia	Porphyria Rare Disease Clinical Research	PRISM	question	CAP to provide	en	

experienced because of sun sensitivity?					Porphyria (EP)			to sun sensitivity			Consortium					
Is thickened skin on your hands a chronic skin change you have experienced because of sun sensitivity?	☐ Yes<br>☐ Unknown<br>☐ No		question	Single-select	Disorder-Specific Information: Erythropoietic Porphyria (EP)	patient	02/15/2011	Yes/no indicator to ask if the patient has thickened skin on hands due to sun sensitivity	thickened skin on hands indicator	skin manifestations; photophobia	Porphyria Rare Disease Clinical Research Consortium	PRISM	question	CAP to provide	en	
Is thickened skin on your feet a chronic skin change you have experienced because of sun sensitivity?	☐ Yes<br>☐ Unknown<br>☐ No		question	Single-select	Disorder-Specific Information: Erythropoietic Porphyria (EP)	patient	02/15/2011	Yes/no indicator to ask if the patient has thickened skin on feet due to sun sensitivity	thickened skin on feet indicator	skin manifestations; photophobia	Porphyria Rare Disease Clinical Research Consortium	PRISM	question	CAP to provide	en	
Is thickened skin on your face a chronic skin change you have experienced because of sun sensitivity?	☐ Yes<br>☐ Unknown<br>☐ No		question	Single-select	Disorder-Specific Information: Erythropoietic Porphyria (EP)	patient	02/15/2011	Yes/no indicator to ask if the patient has thickened skin on face due to sun sensitivity	thickened skin on face indicator	skin manifestations; photophobia	Porphyria Rare Disease Clinical Research Consortium	PRISM	question	CAP to provide	en	
Is thickened skin on your ears a	☐ Yes<br>☐ Unknown<br>☐ No		question	Single-select	Disorder-Specific Informat	patient	02/15/2011	Yes/no indicator to ask if the	thickened skin on ears indicator	skin manifestations;	Porphyria a Rare Disease	PRISM	question	CAP to prov	en	

chronic skin change you have experienced because of sun sensitivity?					ion: Erythropoietic Porphyria (EP)			patient has thickened skin on ears due to sun sensitivity		photophobia	Clinical Research Consortium			ide		
Is thickened skin on your legs a chronic skin change you have experienced because of sun sensitivity?	☐ Yes<br>☐ Unknown<br>☐ No		question	Single-select	Disorder-Specific Information: Erythropoietic Porphyria (EP)	patient	02/15/2011	Yes/no indicator to ask if the patient has thickened skin on legs due to sun sensitivity	thickened skin on legs indicator	skin manifestations; photophobia	Porphyria Rare Disease Clinical Research Consortium	PRISM	question	CAP to provide	en	
Is thickened skin on your arms a chronic skin change you have experienced because of sun sensitivity?	☐ Yes<br>☐ Unknown<br>☐ No		question	Single-select	Disorder-Specific Information: Erythropoietic Porphyria (EP)	patient	02/15/2011	Yes/no indicator to ask if the patient has thickened skin on arms due to sun sensitivity	thickened skin on arms indicator	skin manifestations; photophobia	Porphyria Rare Disease Clinical Research Consortium	PRISM	question	CAP to provide	en	
Is cracking on your hands a chronic skin change you have experienced because of sun sensitivity?	☐ Yes<br>☐ Unknown<br>☐ No		question	Single-select	Disorder-Specific Information: Erythropoietic Porphyria (EP)	patient	02/15/2011	Yes/no indicator to ask if the patient has cracking on hands due to sun sensitivity	cracking on hands indicator	Judge Misch Wright Syndrome	Porphyria Rare Disease Clinical Research Consortium	PRISM	question	CAP to provide	en	

A Sample of PRISM Questions and Selected Metadata

Is cracking on your feet a chronic skin change you have experienced because of sun sensitivity?	☐ Yes<br>☐ Unknown<br>☐ No		question	Single-select	Disorder-Specific Information: Erythropoietic Porphyria (EP)	patient	02/15/2011	Yes/no indicator to ask if the patient has cracking on feet due to sun sensitivity	cracking on feet indicator	Judge Misch Wright Syndrome	Porphyria Rare Disease Clinical Research Consortium	PRISM	question	CAP to provide	en	
Is cracking on your face a chronic skin change you have experienced because of sun sensitivity?	☐ Yes<br>☐ Unknown<br>☐ No		question	Single-select	Disorder-Specific Information: Erythropoietic Porphyria (EP)	patient	02/15/2011	Yes/no indicator to ask if the patient has cracking on face due to sun sensitivity	cracking on face indicator	Judge Misch Wright Syndrome	Porphyria Rare Disease Clinical Research Consortium	PRISM	question	CAP to provide	en	
Is cracking on your ears a chronic skin change you have experienced because of sun sensitivity?	☐ Yes<br>☐ Unknown<br>☐ No		question	Single-select	Disorder-Specific Information: Erythropoietic Porphyria (EP)	patient	02/15/2011	Yes/no indicator to ask if the patient has cracking on ears due to sun sensitivity	cracking on ears indicator	Judge Misch Wright Syndrome	Porphyria Rare Disease Clinical Research Consortium	PRISM	question	CAP to provide	en	
Is cracking on your legs a chronic skin change you have experienced because of sun sensitivity?	☐ Yes<br>☐ Unknown<br>☐ No		question	Single-select	Disorder-Specific Information: Erythropoietic Porphyria (EP)	patient	02/15/2011	Yes/no indicator to ask if the patient has cracking on legs due to sun sensitivity	cracking on legs indicator	Judge Misch Wright Syndrome	Porphyria Rare Disease Clinical Research Consortium	PRISM	question	CAP to provide	en	
Is cracking	☐ Yes		ques	Single-	Disorder	pati	02/15	Yes/no	cracking on	Judge	Porphyri	PRI	ques	CAP	e	

on your arms a chronic skin change you have experienced because of sun sensitivity?	☐ Unknown<br>☐ No		tion	select	-Specific Information: Erythropoietic Porphyria (EP)	ent	/2011	indicator to ask if the patient has cracking on arms due to sun sensitivity	arms indicator	Misch Wright Syndrome	a Rare Disease Clinical Research Consortium	SM	tion	to provide	n	
Are pigment changes on your hands a chronic skin change you have experienced because of sun sensitivity?	☐ Yes<br>☐ Unknown<br>☐ No		question	Single-select	Disorder-Specific Information: Erythropoietic Porphyria (EP)	patient	02/15/2011	Yes/no indicator to ask if the patient has pigment changes on hands due to sun sensitivity	pigment changes on hands indicator	photophobia; skin manifestations	Porphyria Rare Disease Clinical Research Consortium	PRISM	question	CAP to provide	en	
Are pigment changes on your feet a chronic skin change you have experienced because of sun sensitivity?	☐ Yes<br>☐ Unknown<br>☐ No		question	Single-select	Disorder-Specific Information: Erythropoietic Porphyria (EP)	patient	02/15/2011	Yes/no indicator to ask if the patient has pigment changes on feet due to sun sensitivity	pigment changes on feet indicator	photophobia; skin manifestations	Porphyria Rare Disease Clinical Research Consortium	PRISM	question	CAP to provide	en	
Are pigment changes on your face a chronic skin change you have experienced because of sun	☐ Yes<br>☐ Unknown<br>☐ No		question	Single-select	Disorder-Specific Information: Erythropoietic Porphyria (EP)	patient	02/15/2011	Yes/no indicator to ask if the patient has pigment changes on face due to sun sensitivity	pigment changes on face indicator	photophobia; skin manifestations	Porphyria Rare Disease Clinical Research Consortium	PRISM	question	CAP to provide	en	

sensitivity?																
Are pigment changes on your ears a chronic skin change you have experienced because of sun sensitivity?	Yes<br>Unknown<br>No		question	Single-select	Disorder-Specific Information: Erythropoietic Porphyria (EP)	patient	02/15/2011	Yes/no indicator to ask if the patient has pigment changes on ears due to sun sensitivity	pigment changes on ears indicator	photophobia; skin manifestations	Porphyria Rare Disease Clinical Research Consortium	PRISM	question	CAP to provide	en	
If you have acute hepatic porphyria (ALAD, AIP, HCP or VP), please indicate all your symptoms	Severe abdominal pain<br>Neurological symptoms<br>Other (specify)<br>Skin photosensitivity<br>No symptoms<br>Psychiatric symptoms		question	multi select	Disease symptoms	patient		Type of symptoms from enumerated list	Symptoms indicator	porphyrias	Porphyria Rare Disease Clinical Research Consortium	PRISM	question	CAP to provide	en	
If you indicated skin photosensitivity for your symptoms of acute hepatic	Redness<br>Scarring<br>Swelling<br>Blistering		question	single select	Disease symptoms	patient	01/10/2011	Type of skin photosensitivity from enumerated list	Skin photosensitivity indicator	porphyrias; photosensitivity disorders	Porphyria Rare Disease Clinical Research Consortium	PRISM	question	CAP to provide	en	

A Sample of PRISM Questions and Selected Metadata

porphyria, please specify type																
If you have cutaneous porphyria (CEP, PCT, EPP, XLP), please indicate your symptoms	☐ Skin sensitive to sun<br>☐ No symptoms<br>☐ Liver complications		question	multi select	Disease symptoms	patient	01/10/2011	Symptoms from enumerated list	Symptoms indicator	porphyrias	Porphyria Rare Disease Clinical Research Consortium	PRISM	question	CAP to provide	en	
If you indicated skin photosensitivity for your symptoms of cutaneous porphyria, please specify type	☐ Redness<br>☐ Scarring<br>☐ Swelling<br>☐ Blistering		question	single select	Disease symptoms	patient	01/10/2011	Type of photosensitivity from enumerated list	Skin photosensitivity indicator	porphyrias; photosensitivity disorders	Porphyria Rare Disease Clinical Research Consortium	PRISM	question	CAP to provide	en	
Did the iron supplement affect sun sensitivity?	☐ Better<br>☐ Unknown<br>☐ Unchanged<br>☐ Worse		question	Single-select	Disorder-Specific Information: Erythropoietic Porphyria (EP)	patient	02/15/2011		effect of iron supplement on sun sensitivity	photophobia; anemia	Porphyria Rare Disease Clinical Research Consortium	PRISM	question	CAP to provide	en	
Have you had a bone density study?	☐ Yes<br>☐ Unknown<br>☐ No		question	Single-select	Disorder-Specific Information: Erythropoietic Porphyri	patient	02/15/2011	Yes/no indicator to ask if the patient ever had a bone density study	bone density study indicator	tomography, x-ray computed; bone	Porphyria Rare Disease Clinical Research Consorti	PRISM	question	CAP to provide	en	

A Sample of PRISM Questions and Selected Metadata

					a (EP)						um					
What was the date of your last bone density study?	☐ Unknown<br>☐ Number field	date	question	Date (dd-mm-yyyy)	Disorder-Specific Information: Erythropoietic Porphyria (EP)	patient	02/15/2011	Date (dd/mm/yyyy) specifying date of last bone density study	date of bone density study	tomography, x-ray computed; bone	Porphyria Rare Disease Clinical Research Consortium	PRISM	question	CAP to provide	en	
What were the findings of your bone density study?	☐ Unknown<br>☐ Text field	text	question	Text	Disorder-Specific Information: Erythropoietic Porphyria (EP)	patient	02/15/2011	Text specifying findings of bone density study	findings of bone density study	tomography, x-ray computed; bone	Porphyria Rare Disease Clinical Research Consortium	PRISM	question	CAP to provide	en	
Have you been diagnosed with Congenital Erythropoietic Porphyria (CEP)?	☐ Yes<br>☐ Unknown<br>☐ No		question	single select	Disease specific medical history	patient	01/10/2011	Yes/no indicator to ask if patient has been diagnosed with Congenital Erythropoettic Porphyria (Porphyria refers to a group of disorders that result in a buildup of chemicals called porphyrins in your body. Although porphyrins	Congenital Erythropoietic porphyria diagnosis indicator	porphyrias	Porphyria Rare Disease Clinical Research Consortium	PRISM	question	CAP to provide	en	

								are normal body chemicals, i								
Did you develop ulcerations of the skin at sites of blisters?	☐ Yes<br>☐ Unknown<br>☐ No		question	Single-select	Disorder-Specific Information: Porphyria Cutanea Tarda	patient	02/08/2011	Yes/no indicator to ask if a patient's symptoms included ulcerations of the skin at sites of blisters	skin symptom of ulcerations of the skin at sites of blisters indicator	Ulcer	Porphyria Rare Disease Clinical Research Consortium	PRISM	question	CAP to provide	en	
Did you develop infections of the skin at sites of blisters?	☐ Yes<br>☐ Unknown<br>☐ No		question	Single-select	Disorder-Specific Information: Porphyria Cutanea Tarda	patient	02/08/2011	Yes/no indicator to ask if a patient's symptoms included infections of the skin at sites of blisters	skin symptom of infections of the skin at sites of blisters indicator	Infection	Porphyria Rare Disease Clinical Research Consortium	PRISM	question	CAP to provide	en	
Have you ever been diagnosed with a malignancy (excluding dysplasia)?	☐ Yes, one site<br>☐ Unknown<br>☐ No<br>☐ Yes, more than one site		question	single-select	Medical history	patient	02/14/2011	Yes/no indicator to ask if patient was diagnosed with a malignancy excluding dysplasia (new abnormal growth of cancerous tissue)	malignancy (excluding dysplasia) indicator	neoplasm	Rare Thrombosis Disease Network	PRISM	question	Assigned by CAP	en	

A Sample of PRISM Questions and Selected Metadata

Did you develop scars at sites of water blisters or peeled off skin?	☐ Yes<br>☐ Unknown<br>☐ No		question	Single-select	Disorder-Specific Information: Porphyria Cutanea Tarda	patient	02/08/2011	Yes/no indicator to ask if a patient's symptoms included scars at sites of water blisters or peeled off skin	skin symptom of scars at sites of water blisters or peeled off skin indicator	Cicatrix	Porphyria Rare Disease Clinical Research Consortium	PRISM	question	CAP to provide	en	
	☐ Hole in the heart (ventricular or atrial septal defect)<br>☐ Crossed artery (transposition)<br>☐ Other (please specify)<br>☐ Slow heart beat (congenital or complete heart block)<br>☐ Abnormal valves (mitral, tricuspid, aortic,		question	multi-select		patient	01/31/2011	Enumerated list to indicate cardiac abnormalities	Perinatal History	pediatrics; heart; abnormalities	Vasculitis Reproductive Health Questionnaire	RDCRN	question	URI	en	

	pulmonic valves)															
Have you ever been tested for Factor V Leiden?	☐ Not done<br>☐ Done, negative (normal)<br>☐ Unknown<br>☐ Done, positive (homozygous)<br>☐ Done, positive (heterozygous)		question	single-select	Medical history	patient	02/14/2011	Yes/no indicator to ask if patient has been trested for Factor V Leiden (activated C Protein resistance)	Factor V Leiden test indicator	factor V Leiden	Rare Thrombosis Disease Network	PRISM	question	Assigned by CAP	en	
Have you ever been tested for Prothrombin G20210A?	☐ Not done<br>☐ Done, negative (normal)<br>☐ Unknown<br>☐ Done, positive (homozygous)<br>☐ Done, positive (heterozygous)		question	single-select	Medical history	patient	02/14/2011	Yees/no indicator to ask if patient was ever tested for prothrombin G20210A (prothrombins are precursors of thrombin)	Prothrombin G20210A test indicator	prothrombin	Rare Thrombosis Disease Network	PRISM	question	Assigned by CAP	en	
All people have two hemochromatosis genes. Did you have a gene test (DNA test) to see if you had an	☐ Yes<br>☐ Unknown<br>☐ No		question	Single-select	Disorder-Specific Information: Porphyria Cutanea Tarda	patient	02/08/2011	Yes/no indicator to ask if patient had a gene test (DNA test) to see if he/she had an inherited disorder	gene test for hemochromatosis indicator	Hemochromatosis	Porphyria Rare Disease Clinical Research Consortium	PRISM	question	CAP to provide	en	

inherited disorder called hemochromatosis (A disorder due to the deposition of hemosiderin in the parenchymal cells, causing tissue damage and dysfunction								called hemochromatosis, which is a disorder due to the deposition of hemosiderin in the parenchymal cells, causing tissue damage and dysfunction of th								
What was the result of your liver inflammation test?	Positive<br>Unknown<br>Negative		question	Single-select	Disorder-Specific Information: Porphyria Cutanea Tarda	patient	02/08/2011	Indicator to ask if patient had a positive or negative result on his/her liver inflammation test	liver inflammation test result indicator	Clinical Chemistry Tests; Hepatitis	Porphyria Rare Disease Clinical Research Consortium	PRISM	question	CAP to provide	en	
Have you ever been tested for Hepatitis C?	Not done<br>Unknown<br>Done, positive<br>Done, negative		question	single-select	medical testing	patient	02/14/2011	Yes/no indicator to ask if patientn was ever tested for Hepatitis C, an inflammation of the liver caused by Hepatitis C virus, often associated	Hepatitic C test indicator	hepatitis C	Rare Thrombosis Disease Network	PRISM	question	Assigned by CAP	en	

								with transfusions and intravenous drug abuse.								
Have you ever been tested for CMV Serology?	Not done<br>Unknown<br>Done, positive<br>Done, negative		question	single-select	medical testing	patient	02/14/2011	Yes/no indicator to ask if patient was tested for CMC serology (cytomagalovirus infects many organs and is an opportunistic infection in AIDS	CMV Serology test indicator	cytomegalovirus	Rare Thrombosis Disease Network	PRISM	question	Assigned by CAP	en	
What was the result of your hepatitis C test?	Positive<br>Unknown<br>Negative		question	Single-select	Disorder-Specific Information: Porphyria Cutanea Tarda	patient	02/08/2011	Indicator to ask if patient had a positive or negative result on his/her hepatitis C virus test	hepatitis C test result indicator	Clinical Chemistry Tests; Hepatitis C	Porphyria Rare Disease Clinical Research Consortium	PRISM	question	CAP to provide	en	
Did your liver biopsy sample show excess fat deposits in liver cells?	Yes<br>Unknown<br>No		question	Single-select	Disorder-Specific Information: Porphyria Cutanea Tarda	patient	02/08/2011	Yes/no indicator to ask if the patient's liver biopsy sample showed excess fat deposits in liver cells. Hepatocytes	liver biopsy showing excess fat deposits in liver cells indicator	Liver; Biopsy; Hepatocytes	Porphyria Rare Disease Clinical Research Consortium	PRISM	question	CAP to provide	en	

								are the main structural component of the liver. They are specialized epithelial cells that are organized into interconnected plates called								
	☐ Pill<br>☐ IUD (intrauterine device)<br>☐ Patch<br>☐ Diaphragm<br>☐ Condom<br>☐ Ring<br>☐ Other (please specify)<br>☐ Spermicides<br>☐ Tubal ligation (tubes tied)<br>☐ Withdrawal<br>☐ Rhythm or natural method		question	multi-select		patient	01/31/2011	Enumerated list of contraception methods	Contraception	contraception	Vasculitis Reproductive Health Questionnaire	RDCRN	question	URI	en	

	☐ Partner sterilized (vasectomy)<br>☐ Abstinence (not having sex)<br>☐ Shot (Depo-Provera)															
Do you have mouth ulcers/mucositis?	☐ Yes<br>☐ Unknown<br>☐ No		question	single-select	Disease specific medical history	patient	02/08/2011	Yes/no indicator to ask if patient has mouth ulcers/mucositis (mucositis is an inflammation of the mucosa with burning or tingling sensation, and usually occurs at the mucous lining of the mouth, the gastrointestinal tract or the airway due to chemical ir	mouth ulcer/mucositis indicator	mucositis; oral ulcers	Rare Lung Diseases Consortium	PRISM	question	Assigned by CAP	en	
What was the result of your human	☐ Positive<br>☐ Unknown<br>☐ Negative		question	Single-select	Disorder-Specific Informat	patient	02/08/2011	Indicator to ask if patient had a	HIV test result indicator	Clinical Chemistry Tests;	Porphyria Rare Disease	PRISM	question	CAP to prov	en	

immunodeficiency virus (HIV) test?					ion: Porphyria Cutanea Tarda			positive or negative result on his/her human immunodeficiency virus (HIV) test		HIV	Clinical Research Consortium			ide		
Sitting or lying still	☐ FALSE<br>☐ TRUE		scale	single-select	Disease specific medical history	patient	01/20/2011	True/false indicator to ask if sitting or lying still makes patient feel shortness of breath (difficult of labored breathing)	Patient report of shortness of breath triggers	Dyspnea	St. George's Medical School, London	PRISM	scale	URI	en	P.W. Jones, PhD., FRCP, Prof. of Respiratory Medicine, St. George's Hospital Medical School
If your Hepatitis C test was positive, were you treated with interferon?	☐ Yes<br>☐ Unknown<br>☐ No		question	Single-select	Disorder-Specific Information: Porphyria Cutanea Tarda	patient	02/08/2011	Yes/no indicator to ask if the patient was treated with interferon if his/her Hepatitis C test was positive	interferon treatment indicator	Interferons; Hepatitis C	Porphyria Rare Disease Clinical Research Consortium	PRISM	question	CAP to provide	en	
If your Hepatitis C test was positive, were you treated with interferon and ribavarin?	☐ Yes<br>☐ Unknown<br>☐ No		question	Single-select	Disorder-Specific Information: Porphyria Cutanea Tarda	patient	02/08/2011	Yes/no indicator to ask if the patient was treated with interferon and ribavarin if his/her Hepatitis C	interferon and ribavarin treatment indicator	Interferons; Ribavirin; Hepatitis C	Porphyria Rare Disease Clinical Research Consortium	PRISM	question	CAP to provide	en	

A Sample of PRISM Questions and Selected Metadata

								test was positive								
Visiting family or friends or playing with children			scale	Blank field	Lifestyle	patient	01/20/2011	Statement indicating that lung/respiratory problem may prevent patient from visiting family, friends, or playing with children (pathological processes involving any part of the lung)	Information for patient	Lung Diseases	St. George's Medical School, London	PRISM	scale	URI	en	P.W. Jones, PhD., FRCP, Prof. of Respiratory Medicine, St. George's Hospital Medical School
Have you had a bone density test (scan)?	☐ Yes<br>☐ Unknown<br>☐ No		question	single-select	Other tests	patient	01/24/2010	Yes/no indicator to ask whether a patient has had a bone density test.	bone density test indicator	Absorptiometry, Photon	Osteogenesis Imperfecta Foundation	PRISM	question		en	
	☐ Yes<br>☐ Unknown<br>☐ No															
Has sun sensitivity changed as you have gotten older?	☐ Yes<br>☐ Unknown<br>☐ No		question	Single-select	Disorder-Specific Information: Congenital Erythropoietic Porphyri	patient	02/15/2011	Yes/no indicator to ask if sun sensitivity changed as the patient has gotten older	sun sensitivity change with age indicator	photophobia; signs and symptoms	Porphyria Rare Disease Clinical Research Consortium	PRISM	question	CAP to provide	en	

					a (CEP)											
How has your sun sensitivity changed as you have gotten older?	☐ It has worsened<br>☐ Unknown<br>☐ It has improved		question	Single-select	Disorder-Specific Information: Congenital Erythropoietic Porphyria (CEP)	patient	02/15/2011	Enumerated list to indicate how the patient's sun sensitivity has changed as he/she has gotten older	degree of sun sensitivity change with age indicator	photophobia; signs and symptoms	Porphyria Rare Disease Clinical Research Consortium	PRISM	question	CAP to provide	en	
If you have had children, did your sun sensitivity change during pregnancy?	☐ Yes<br>☐ Unknown<br>☐ Never been pregnant<br>☐ No		question	Single-select	Disorder-Specific Information: Congenital Erythropoietic Porphyria (CEP)	patient	02/15/2011	Yes/no indicator to ask if a female patient with children experienced a change in sun sensitivity during pregnancy	sun sensitivity change during pregnancy indicator	pregnancy; photophobia; signs and symptoms	Porphyria Rare Disease Clinical Research Consortium	PRISM	question	CAP to provide	en	
How did your sun sensitivity change during your pregnancy?	☐ It has worsened<br>☐ Unknown<br>☐ Never been pregnant<br>☐ It has improved		question	Single-select	Disorder-Specific Information: Congenital Erythropoietic Porphyria (CEP)	patient	02/15/2011	Enumerated list to indicate how a female patient's sun sensitivity changed during pregnancy	degree of sun sensitivity change during pregnancy indicator	pregnancy; photophobia; signs and symptoms	Porphyria Rare Disease Clinical Research Consortium	PRISM	question	CAP to provide	en	
Do you have sensitivity to indoor (fluorescent)	☐ Yes<br>☐ Unknown<br>☐ No		question	Single-select	Disorder-Specific Information:	patient	02/15/2011	Yes/no indicator to ask if a patient is	indoor lighting sensitivity indicator	photophobia; signs and symptom	Porphyria Rare Disease Clinical	PRISM	question	CAP to provide	en	

lighting?					Congenital Erythropoietic Porphyria (CEP)			sensitive to indoor (fluorescent) lighting		s	Research Consortium					
Do you experience sun sensitivity or rashes as an active symptom of your acute porphyria?	☐ Yes<br>☐ Unknown<br>☐ No		question	Single-select	Disorder-Specific Information: Acute Porphyria (Acute Intermittent Porphyria, Hereditary Coproporphyria, Variegate Porphyria)	patient	02/08/2011	Yes/no indicator to ask if the patient experiences sun sensitivity or rashes as an active symptom of acute porphyria	sun sensitivity or rashes as acute porphyria symptom indicator	exanthema; photophobia; porphyrias, acute intermittent	Porphyria Rare Disease Clinical Research Consortium	PRISM	question	CAP to provide	en	
Have you ever been diagnosed with Multiple Chemical Sensitivity?	☐ Yes<br>☐ Unknown<br>☐ No		question	Single-select	Disorder-Specific Information: Acute Porphyria (Acute Intermittent Porphyria, Hereditary	patient	02/08/2011	Yes/no indicator to ask if the patient has ever been diagnosed with Multiple Chemical Sensitivity, an acquired disorder characterized by	Multiple Chemical Sensitivity indicator	multiple chemical sensitivity	Porphyria Rare Disease Clinical Research Consortium	PRISM	question	CAP to provide	en	

					Coproporphyria, Variegate Porphyria)			recurrent symptoms, referable to multiple organ systems, occurring in response to demonstrable exposure to many chemic								
Have you experienced reduced bone density related to Congenital Erythropoietic Porphyria (CEP)?	☐ Yes<br>☐ Unknown<br>☐ No		question	Single-select	Disorder-Specific Information: Congenital Erythropoietic Porphyria (CEP)	patient	02/15/2011	Yes/no indicator to ask if the patient has experienced reduced bone density related to Congenital Erythropoietic Porphyria (CEP)	reduced bone density related to Congenital Erythropoietic Porphyria (CEP) indicator	bone diseases; signs and symptoms; porphyria, erythropoietic	Porphyria Rare Disease Clinical Research Consortium	PRISM	question	CAP to provide	en	
Have you avoided outdoor walking/jogging/running during the daytime due to sun sensitivity?	☐ Yes<br>☐ Unknown<br>☐ No		question	Single-select	Disorder-Specific Information: Congenital Erythropoietic Porphyria (CEP)	patient	02/15/2011	Yes/no indicator to ask if the patient has avoided outdoor walking/jogging/running during the daytime due to sun sensitivity	avoidance of outdoor walking/jogging/running during the daytime indicator	photophobia; health behavior	Porphyria Rare Disease Clinical Research Consortium	PRISM	question	CAP to provide	en	
Have you avoided	☐ Yes<br>☐ Unknown		question	Single-select	Disorder-Specific	patient	02/15/2011	Yes/no indicator to	avoidance of driving/ridin	photophobia;	Porphyria Rare	PRISM	question	CAP to	en	

driving/riding in the car during the daytime due to sun sensitivity?	☐ No				Information: Congenital Erythropoietic Porphyria (CEP)			ask if the patient has avoided driving/riding in the car during the daytime due to sun sensitivity	g in the car during the daytime indicator	health behavior	Disease Clinical Research Consortium			provide		
Have you avoided outdoor bicycling during the daytime due to sun sensitivity?	☐ Yes<br>☐ Unknown<br>☐ No		question	Single-select	Disorder-Specific Information: Congenital Erythropoietic Porphyria (CEP)	patient	02/15/2011	Yes/no indicator to ask if the patient has avoided outdoor bicycling during the daytime due to sun sensitivity	avoidance of outdoor bicycling during the daytime indicator	photophobia; health behavior	Porphyria Rare Disease Clinical Research Consortium	PRISM	question	CAP to provide	en	
Have you avoided outdoor shopping during the daytime due to sun sensitivity?	☐ Yes<br>☐ Unknown<br>☐ No		question	Single-select	Disorder-Specific Information: Congenital Erythropoietic Porphyria (CEP)	patient	02/15/2011	Yes/no indicator to ask if the patient has avoided outdoor shopping during the daytime due to sun sensitivity	avoidance of outdoor shopping during the daytime indicator	photophobia; health behavior	Porphyria Rare Disease Clinical Research Consortium	PRISM	question	CAP to provide	en	
Have you avoided outdoor picnics during the daytime due to sun	☐ Yes<br>☐ Unknown<br>☐ No		question	Single-select	Disorder-Specific Information: Congenital Erythrop	patient	02/15/2011	Yes/no indicator to ask if the patient has avoided outdoor picnics	avoidance of outdoor picnics during the daytime indicator	photophobia; health behavior	Porphyria Rare Disease Clinical Research Consorti	PRISM	question	CAP to provide	en	

sensitivity?					oietic Porphyria (CEP)			during the daytime due to sun sensitivity			um					
Have you avoided outdoor parties during the daytime due to sun sensitivity?	☐ Yes<br>☐ Unknown<br>☐ No		question	Single-select	Disorder-Specific Information: Congenital Erythropoietic Porphyria (CEP)	patient	02/15/2011	Yes/no indicator to ask if the patient has avoided outdoor parties during the daytime due to sun sensitivity	avoidance of outdoor parties during the daytime indicator	photophobia; health behavior	Porphyria Rare Disease Clinical Research Consortium	PRISM	question	CAP to provide	en	
Have you avoided outdoor concerts/festivals during the daytime due to sun sensitivity?	☐ Yes<br>☐ Unknown<br>☐ No		question	Single-select	Disorder-Specific Information: Congenital Erythropoietic Porphyria (CEP)	patient	02/15/2011	Yes/no indicator to ask if the patient has avoided outdoor concerts/festivals during the daytime due to sun sensitivity	avoidance of outdoor concerts/festivals during the daytime indicator	photophobia; health behavior	Porphyria Rare Disease Clinical Research Consortium	PRISM	question	CAP to provide	en	
Have you avoided sitting by the window during the daytime due to sun sensitivity?	☐ Yes<br>☐ Unknown<br>☐ No		question	Single-select	Disorder-Specific Information: Congenital Erythropoietic Porphyria (CEP)	patient	02/15/2011	Yes/no indicator to ask if the patient has avoided sitting by the window during the daytime due to sun sensitivity	avoidance of sitting by the window during the daytime indicator	photophobia; health behavior	Porphyria Rare Disease Clinical Research Consortium	PRISM	question	CAP to provide	en	

A Sample of PRISM Questions and Selected Metadata

Have you avoided any other activities during the daytime due to sun sensitivity?	Unknown<br>Text field	text	question	Text	Disorder-Specific Information: Congenital Erythropoietic Porphyria (CEP)	patient	02/15/2011	Text specifying what other activities the patient has avoided during the daytime due to sun sensitivity	avoidance of other activities during the daytime specify	photophobia; health behavior	Porphyria Rare Disease Clinical Research Consortium	PRISM	question	CAP to provide	en	
Did you select your current occupation because of your sun sensitivity?	Yes<br>Unknown<br>Unemployed<br>No		question	Single-select	Disorder-Specific Information: Congenital Erythropoietic Porphyria (CEP)	patient	02/15/2011	Yes/no indicator to ask if patient's selected his/her current occupation due to sun sensitivity	current occupation due to sun sensitivity indicator	photophobia; health behavior	Porphyria Rare Disease Clinical Research Consortium	PRISM	question	CAP to provide	en	
Have you avoided certain outdoor activities because of your sun sensitivity?	Yes, all outdoor activities during the daytime<br>Unknown<br>No<br>Yes, some outdoor activities during the daytime		question	Single-select	Disorder-Specific Information: Congenital Erythropoietic Porphyria (CEP)	patient	02/15/2011	Yes/no indicator to ask if the patient has avoided certain outdoor activities because of his/her sun sensitivity	avoidance of outdoor activities due to sun sensitivity indicator	photophobia; health behavior	Porphyria Rare Disease Clinical Research Consortium	PRISM	question	CAP to provide	en	
Did you experience motor	Yes<br>Not applicabl		Question	single select	Medical History	patient	11/22/2010	Yes/no indicator to ask whether	motor milestone delay		Clinical Investigation of	PRISM	Question	CAP to prov	en	

milestone delay (such as rolling over, sitting up or walking)?	e<br>☐ No							patient experienced motor milestone delay (physical signs of development or maturation of infants and children, such as rolling over, crawling, walking, or sitting up without assistance).	indicator		Neurologic Channelopathies Consortium			ide		
If yes, do you experience any of the following with headaches?	☐ Unilateral pain<br>☐ Throbbing pain<br>☐ Worsening of headache with activity<br>☐ Weakness of one side of your body<br>☐ Vomiting<br>☐ Sensitivity to lights<br>☐ Sensitivit		Question	single select	Disease symptoms	patient	11/22/2010	Enumerated list for type of symptoms that accompany headaches (pain in the cranial region)	headache symptoms type		Clinical Investigation of Neurologic Channelopathies Consortium	PRISM	Question	CAP to provide	en	

	y to sounds<br>Nausea															
